# Assessing the environmental impact of coronary artery bypass grafting to decrease its footprint

**DOI:** 10.1093/ejcts/ezaf054

**Published:** 2025-02-17

**Authors:** Egid M van Bree, Tim Stobernack, Tadzjo Boom, Klaske Geene, Emma J Kooistra, Wilson Li, Evelyn A Brakema, Laura Golsteijn, Pleun Hemelaar, Scott McAlister, Forbes McGain, Rosalie van Zelm, Hugo R W Touw

**Affiliations:** Department of Intensive Care Medicine, Radboudumc, Nijmegen, Netherlands; Department of Surgery, Maastricht University, Maastricht, Netherlands; Department of Intensive Care Medicine, Radboudumc, Nijmegen, Netherlands; Department of Intensive Care Medicine, Radboudumc, Nijmegen, Netherlands; Department of Intensive Care Medicine, Radboudumc, Nijmegen, Netherlands; Department of Intensive Care Medicine, Radboudumc, Nijmegen, Netherlands; Department of Cardiothoracic Surgery, Radboudumc, Nijmegen, Netherlands; Department of Public Health and Primary Care, Leiden University Medical Center, Leiden, Netherlands; PRé Sustainability, Amersfoort, Netherlands; Department of Intensive Care Medicine, Radboudumc, Nijmegen, Netherlands; Department of Critical Care, University of Melbourne, Melbourne, Australia; Department of Critical Care, University of Melbourne, Melbourne, Australia; Department of Anaesthesia and Intensive Care, Western Health, Melbourne, Australia; Department of Environmental Sciences, Radboud University, Nijmegen, Netherlands; Department of Intensive Care Medicine, Radboudumc, Nijmegen, Netherlands

**Keywords:** Carbon footprint, Life cycle assessment, Sustainable healthcare, Greenhouse gases, Cardiac surgery

## Abstract

**OBJECTIVES:**

An urgent transition to environmentally sustainable healthcare is required. The purpose of this study was to identify key areas for environmental impact mitigation for a coronary artery bypass grafting trajectory.

**METHODS:**

An ISO14040/44 standardized life cycle assessment was conducted for the functional unit of an individual patient trajectory of elective coronary artery bypass grafting surgery, from operating room admission until intensive care unit discharge. Data were collected for products, processes, and services required for care delivery in a Dutch academic hospital for 12 patients. The environmental impact was calculated using the ReCiPe 2016 method.

**RESULTS:**

A single patient trajectory caused 414 [IQR 383–461] kgCO_2_ equivalents of global warming, equal to 2753 km of driving an average Dutch petrol-fuelled car. Other notable environmental impacts were fine particulate matter, (non-)carcinogenic toxicity, land use, and terrestrial acidification. Operating room disposable products (162 kgCO_2_eq), energy use (48 kgCO_2_eq), and employee commute (36 kgCO_2_eq) contributed most to global warming. The extracorporeal circulation set, surgical drapes, intraoperative salvage set, surgical gowns, and cotton gauzes caused most of the disposables’ environmental impact. Most energy use occurred in the operating room via heating, ventilation, and air conditioning.

**CONCLUSIONS:**

A coronary artery bypass grafting trajectory’s environmental impact primarily contributed to global warming. Most impact mitigation could be achieved by avoiding/reducing disposable product use when possible or replacing these with reusables. Optimizing operating room energy systems, switching to renewable energy, and encouraging low-emission employee commute can further reduce the environmental impact.

## GRAPHICAL ABSTRACT

**Figure:**
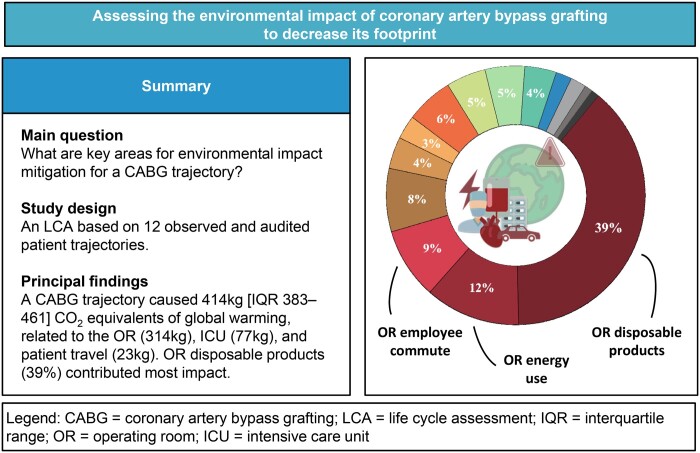

## INTRODUCTION

Human-induced environmental harms, such as climate change and air pollution, have significant implications for global health and require urgent action [1]. The healthcare sector too, contributes to environmental harms, causing 4–8% of CO_2_ emissions in high-income countries [2, 3]. Recent ‘green deals’ in healthcare aim to reduce healthcare’s environmental footprint [4]. To meet these goals, clinicians need to identify and target high-impact areas.

Two studies have previously documented the resource intensity of cardiovascular surgery and reported a wide range in CO_2_ emissions (124–505 kgCO_2_eq) [5, 6]. Methods to estimate associated CO_2_ emissions varied considerably in underlying assumptions, detailedness of data collection, and elements of care considered in their scope [7, 8]. A complete understanding of the environmental impact of cardiovascular surgery, including other impacts than CO_2_ emissions and the postoperative intensive care unit (ICU) admission, is still missing.

Life cycle assessment (LCA) is a routine method to study the environmental impact of products, processes, and services [9]. To date, only an ‘eco audit’ LCA of cardiac surgery has been conducted [5, 7]. The objective of this study, therefore, was to assess a patient trajectory including both coronary artery bypass grafting (CABG) surgery and the postoperative ICU admission to support clinicians in identifying key areas for impact mitigation to deliver more sustainable cardiosurgical care.

## MATERIALS AND METHODS

### Study design

We conducted an LCA of an elective CABG trajectory according to international ISO14040/44 standards [10]. To acquire the necessary clinical data, we performed a single-centre observational study at a Dutch academic hospital. Between 23 May 2022 and 16 June 2022, patients undergoing isolated, on-pump CABG were considered for participation, with a maximum enrolment of one per day. We chose a convenience sample of 12 to allow for sufficient variability in equipment use, operating room (OR) procedure duration, etc.—in line with previous studies [11, 12]. In addition, we performed waste audits for all observed procedures, material studies, desk and literature research, employee interviews, and expert consultations. Where applicable, the Strengthening the Reporting of Observational Studies in Epidemiology (STROBE) criteria were followed. Ethical approval was waived by the Medical Ethical Admission Commission Oost-Nederland (2021-13265). No additional informed consent for participation in the study was collected.

### Life cycle assessment

LCA is a scientific, robust method, using an inventory of material and energy flows (e.g. raw material extraction and creation of plastics) required for a certain product or service (e.g. a surgical gown) to quantify the resulting environmental impacts based on material- and resource-specific characterization factors. According to international standards, LCA consists of four phases: 1) a goal and scope definition; 2) assembly of the inventory relevant to the subject of study; 3) environmental impact assessment, based on a validated model of characterization factors; and 4) interpretation of results, preferably including sensitivity and uncertainty analyses using probability distributions [10, 13]. Further details are provided in Supplementary Material S2.

### Subject and setting

This LCA focussed on isolated CABG surgery specifically, considering that it is the most commonly performed cardiosurgical procedure in the Netherlands (6872–8216 annually in 2019–2021) and globally [14, 15], most frequently on-pump. We concentrated on the resource use and environmental impact of a single type of procedure in order to obtain detailed insights regarding impact mitigation possibilities and to include a quantification of the procedural variability for a relatively homogenous group (not previously reported). Therefore, we excluded combined valve replacement surgeries and off-pump procedures, given their covariant sources of variability. Our academic hospital performed 229–341 CABG surgeries annually in 2017–2021, serving patients directly registered at the hospital and those referred by surrounding peripheral hospitals. The majority of CABG surgeries (±98%) were performed on-pump.

### Data collection

**Figure 1:**
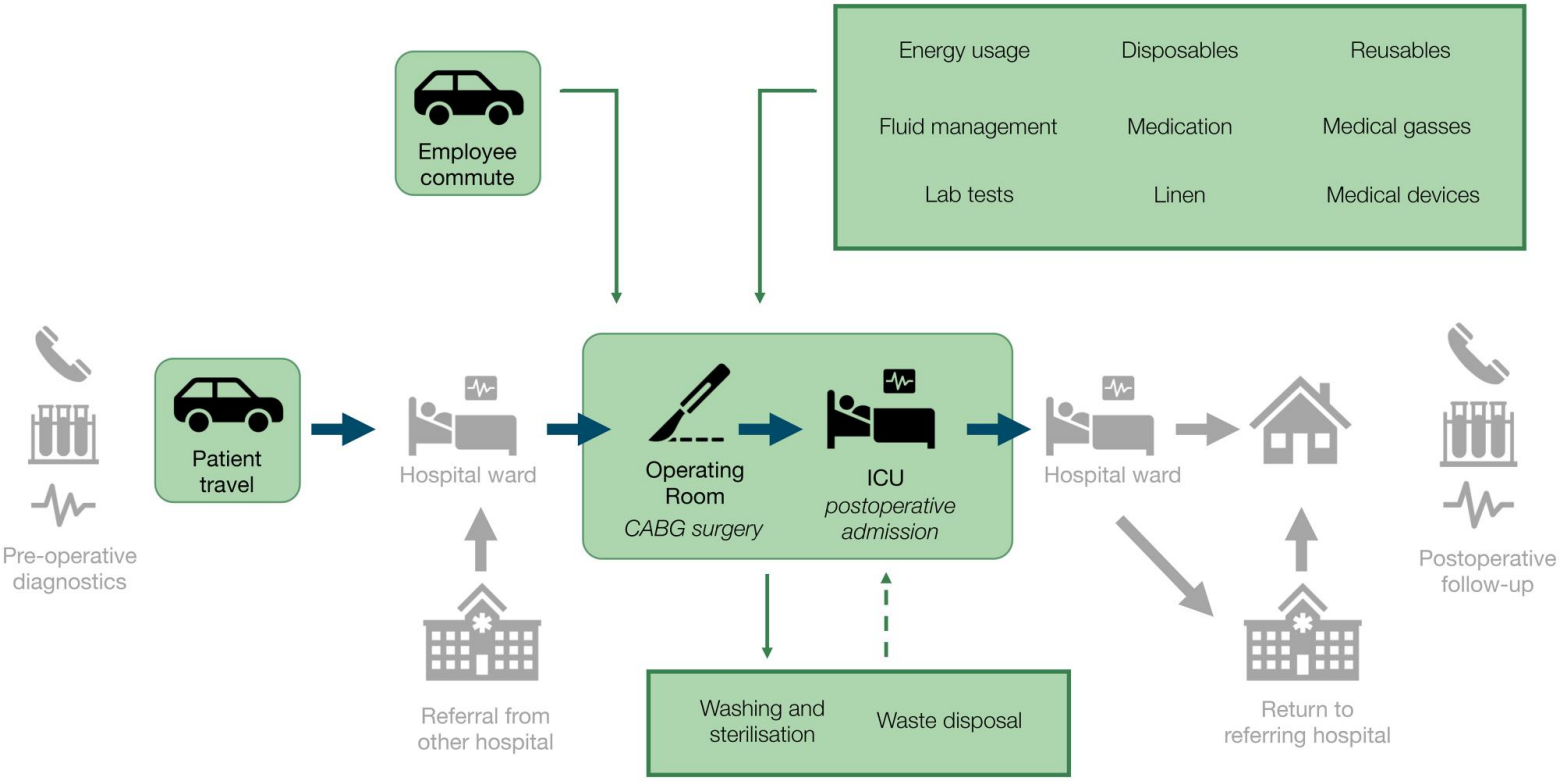
Simplified system boundary of the CABG trajectory. For this LCA: included elements of a CABG trajectory (icons in rounded rectangles), included resources (text in sharp rectangles), and elements not included (icons without a rectangle). Details are reported in Supplementary Material S1. CABG: coronary artery bypass grafting; ICU: intensive care unit; LCA: life cycle assessment.

**Figure 2:**
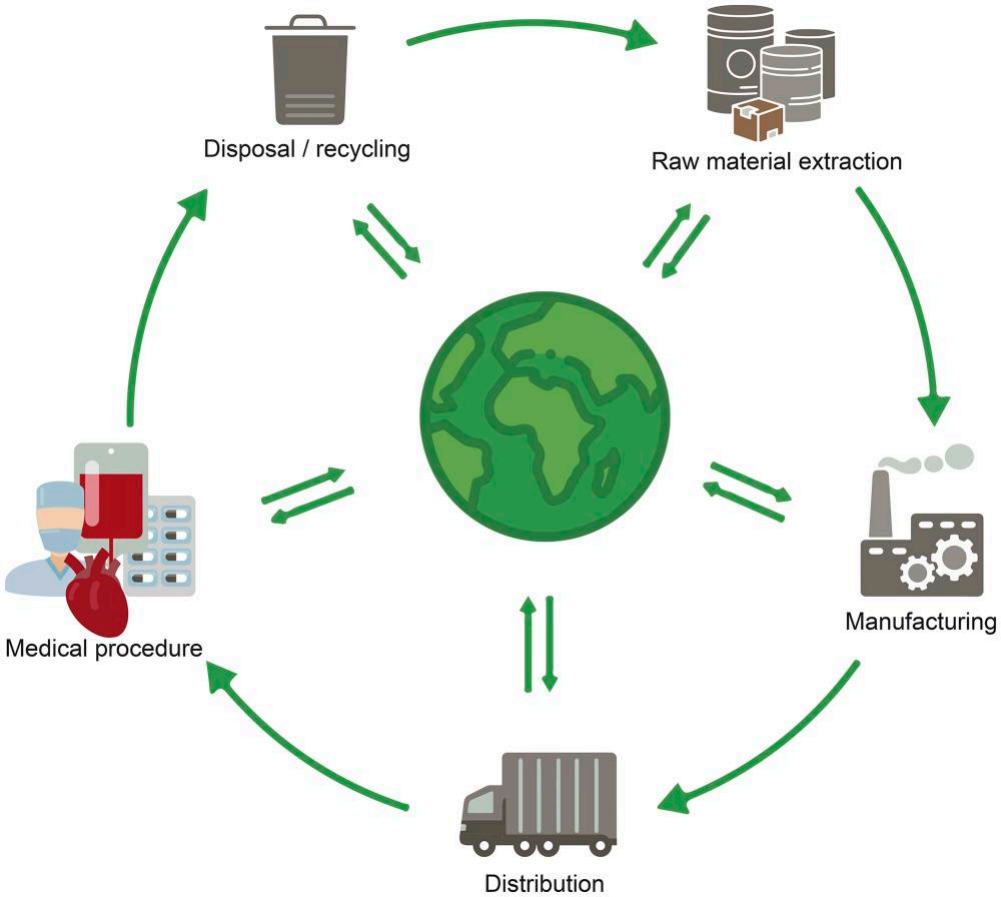
Overview of a CABG trajectory’s life cycle stages. Individual resources required for the CABG trajectory extract substances and materials from the environment and emit others. CABG: coronary artery bypass grafting.

We collected data of required resources per patient, from OR admission until ICU discharge—the LCA’s ‘functional unit’. The elements considered were: disposables, reusables, energy use, employee commute, patient travel, pharmaceuticals, lab tests, fluid management, linen, medical gases, long-standing medical equipment, washing and sterilization, and waste disposal and treatment (Fig. 1, Supplementary Material S1). Hospital infrastructure, consumed food, and room cleaning were not included in the assessment, considering that expected quantities and allocated impacts in the investigated trajectory would be comparatively low. We included data for the entire life cycle, from raw material extraction to disposal or recycling (‘cradle-to-grave’) (Fig. 2). Data were entered into a life cycle inventory in SimaPro LCA software v9.5.0.1 (PRé Sustainability, the Netherlands) and combined with generic ‘background data’ from the ecoinvent v3.9 database (Ecoinvent, Switzerland), containing information on processes such as the creation and incineration of plastics [16]. For electricity and vehicles, more recent national datasets were used [17, 18]. Further details regarding data collection are reported in Supplementary Material S2.

### Study outcomes

We quantified 18 different environmental impact categories and their contributions to human health damage (disability-adjusted life years) and ecosystem damage (species gone extinct over time) using the ReCiPe 2016 v1.1 model of characterization factors, based on environmental and epidemiological scientific consensus [19]. For example, the release of different greenhouse gases and particulate matter incurred by the production and incineration of the polypropylene plastic and polyester of the surgical gowns were aggregated into overarching environmental impact categories, which were in turn characterized by human health and ecosystem damage. We focussed the manuscript’s reporting on the impact categories that individually contributed most damage (≥10%). Outcomes were presented as: (1) absolute values using corresponding ReCiPe reporting units—including kg carbon dioxide equivalents (kgCO_2_eq) for global warming; and (2) a percentage of total human health and ecosystem damage for each of the impact categories.

### Data analysis

We calculated environmental impacts for median values and interquartile ranges [IQR] of observed resource use. Where possible, we used own modelling of resource consumption (e.g. disposables, staff commute, and laundering of linen), complemented by preceding LCA studies if necessary (e.g. sterilization of reusables and pharmaceuticals). A detailed overview is included in Supplementary Material S3. To investigate key areas for impact mitigation, we quantified impacts per group of resources (e.g. OR disposables) to identify the most burdensome products and processes. Due to limited data availability, pharmaceuticals were only considered for the global warming potential caused by active pharmaceutical ingredient production and packaging [11, 20, 21]. To verify the robustness of findings, we performed sensitivity analyses to test the effects of underlying assumptions, database choices, and analysis methods on the identified key areas [13]. In addition, we performed Monte Carlo simulations to compute 95% confidence intervals and analyse uncertainty arising from background data using a pedigree matrix for data quality [22].

### Mitigation scenarios

Upon obtaining the data regarding resource use and environmental impacts, we then considered which of the resource groups were amenable to feasible reductions in a manner that would not impinge upon patient care. We quantified the potential environmental impact reduction for hypothetical scenarios, based on current policies in the investigated hospital (e.g. procuring renewable energy), preceding comparative LCA studies of specific resources (e.g. reusable surgical drapes and gowns), and expert-informed assumptions (e.g. adjustments of the heating, ventilation, and air conditioning (HVAC) system settings). Further details are reported in the corresponding results table in Supplementary Material S6.

## RESULTS

### Procedure descriptives

The median age and European System for Cardiac Operative Risk Evaluation (EuroSCORE) II for included patients were, respectively, 70 [63–74] and 1.5 [IQR 1.0–1.8]. OR duration and ICU length of stay were, respectively, 4.6 [4.2–4.9] and 22.1 [20.0–26.0] h. Patients received total intravenous anaesthesia (TIVA) or balanced anaesthesia and venous grafts were obtained using endoscopic vessel harvesting in the lower limb. CABG surgeries (3–4 anastomoses) were performed by teams of 8–9 team members (surgeon, resident surgeon, anaesthetist, OR nurses, nurse anaesthetist(s), and perfusionist). ICU care was provided by 3.1 full-time equivalents of physicians, nurses, and care support staff. Further procedural details are provided in Supplementary Material S4.

### Resource use

**Figure 3:**
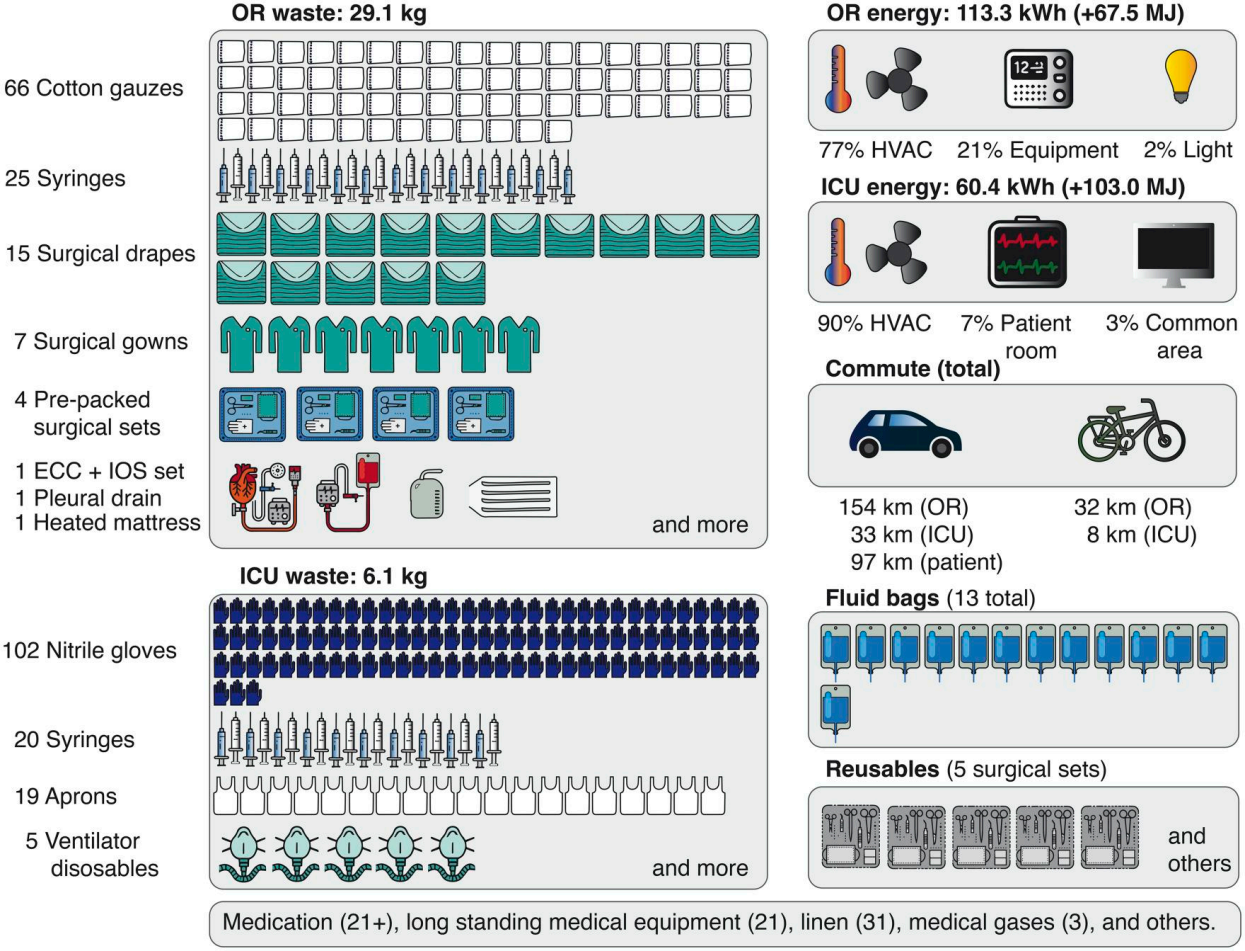
Overview of a CABG trajectory’s resource use. A complete, detailed (numerical) overview of resource use can be found in Supplementary Material S3. CABG: coronary artery bypass grafting; ECC: extracorporeal circulation; ICU: intensive care unit; IOS: intraoperative salvage; OR: operating room.

In total, we identified, categorized, and quantified 561 inputs per patient trajectory. Waste audits resulted in 35.2 [32.1–38.4] kg per trajectory, of which 29.1 [27.1–31.2] kg were collected in the OR and 6.1 [5.0–7.2] kg in the ICU (Fig. 3). By weight, the disposables that contributed most to the trajectory’s waste were: the extracorporeal circulation set (6.4 kg; including polycarbonate blood jars and polyvinyl chloride tubing), surgical drapes (4.8 kg; containing mixtures of viscose, polypropylene, and low-density polyethylene), the intraoperative salvage set (1.5 kg; including a polycarbonate jar and polyvinyl chloride tubing), surgical gowns (0.9–1.0 kg; 7–8 gowns), and cotton gauzes (0.7–0.8 kg; 66–76 pieces). Energy consumption per trajectory amounted to 173.7 [157.6–186.4] kWh of electricity and 170.5 [156.6–191.8] MJ of natural gas, of which 113.3 [102.7–115.4] kWh and 67.5 [63.2–70.4] MJ in the OR, and 60.4 [54.9–71.0] kWh and 103.0 [93.4–121.4] MJ in the ICU. The OR’s HVAC system contributed most to its electricity use (81–90 kWh, 77%). Detailed information regarding resource use for the procedures is available in Supplementary Material S4, and an overview of the life cycle inventory is in Supplementary Material S3, including specifics regarding, e.g. the HVAC system and laundry service.

### Environmental impact

**Figure 4:**
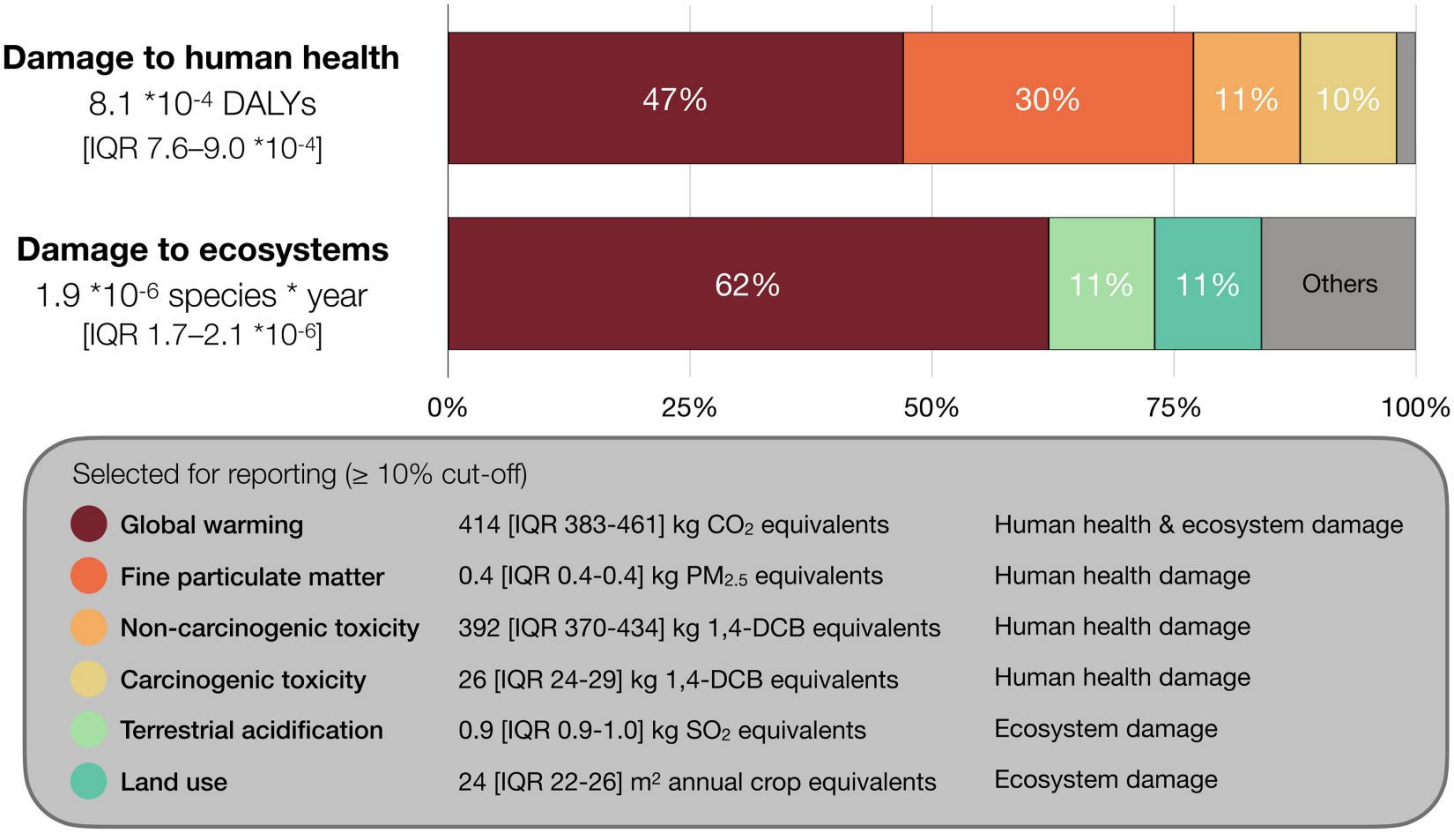
Environmental impact of a CABG trajectory. Percentages are impact categories’ relative contributions to human health damage (DALYs) and/or ecosystem damage, calculated for median values using ReCiPe. Smaller categories not selected for reporting are combined in ‘Others’. CABG: coronary artery bypass grafting; DALYs: disability-adjusted life years.

A single patient CABG trajectory caused 414 [IQR 383–461] kgCO_2_eq of global warming, which contributed most damage to human health (47%) and ecosystems (62%) (Fig. 4). This carbon footprint equals 2753 [2547–3065] km of driving a petrol-fuelled car, compared to emissions whilst driving (tank to wheel) for the average Dutch car [18]. Other environmental impacts contributing ≥10% of damage were: fine particulate matter (0.4 [0.4–0.4] kgPM_2.5_eq; 30%), non-carcinogenic toxicity (392 [370–434] kg 1,4-dichlorobenzene-eq; 11%), and carcinogenic toxicity (26 [24–29] kg 1,4-dichlorobenzene-eq; 10%) for human health; and land use (24 [22–26] m^2^ annual-crop-eq; 11%) and terrestrial acidification (0.9 [0.9–1.0] kgSO_2_eq; 11%) for ecosystems. Further details are reported in Supplementary Material S5.

### Hotspot identification

**Figure 5:**
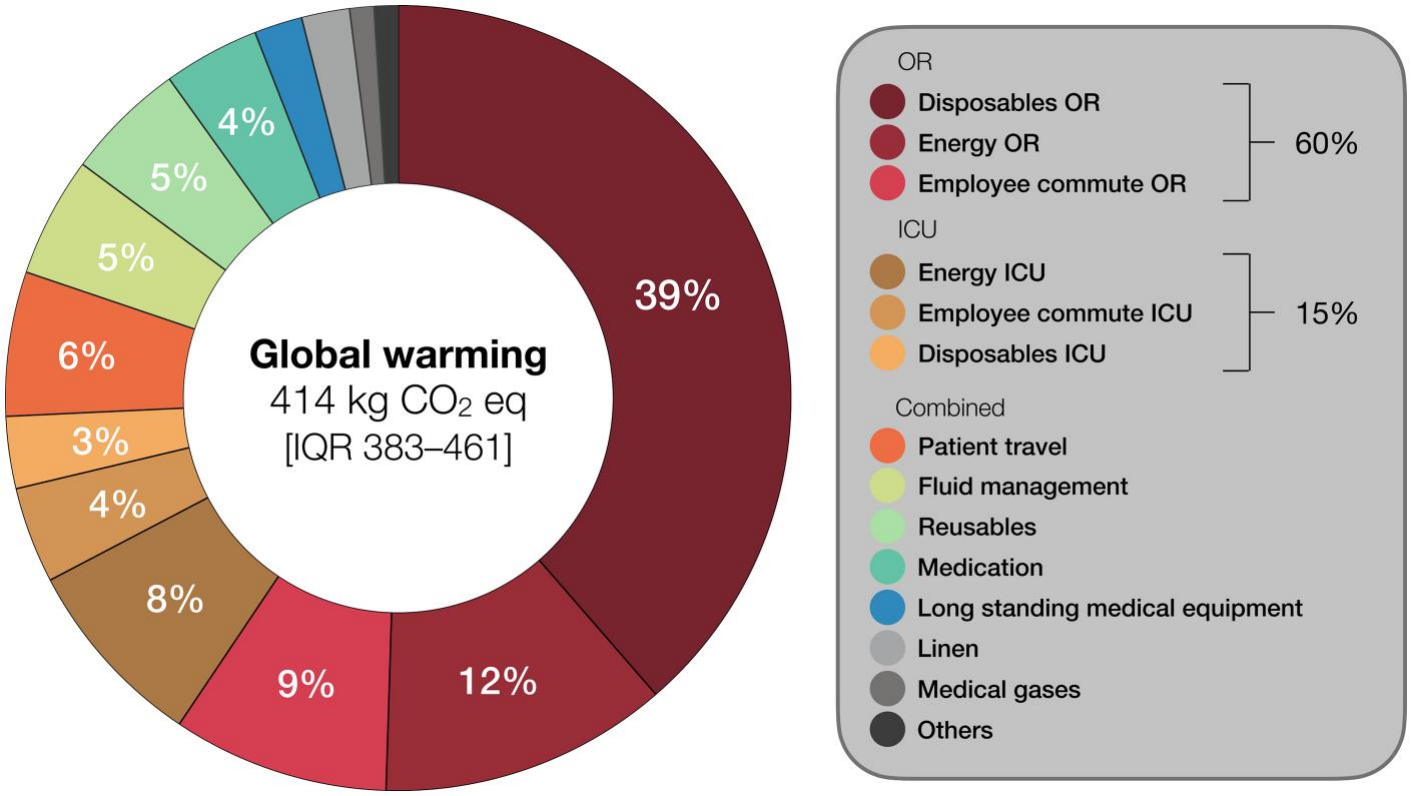
Contribution analysis of a CABG trajectory’s carbon footprint. Percentages are relative contributions to total global warming, calculated for median values (≤2% not indicated). Disposables, energy use, and employee commute are presented separately for OR and ICU; minor resource groups were combined. Anaesthetic gases are included in ‘Medication’. CABG: coronary artery bypass grafting; ICU: intensive care unit; OR: operating room.

**Figure 6:**
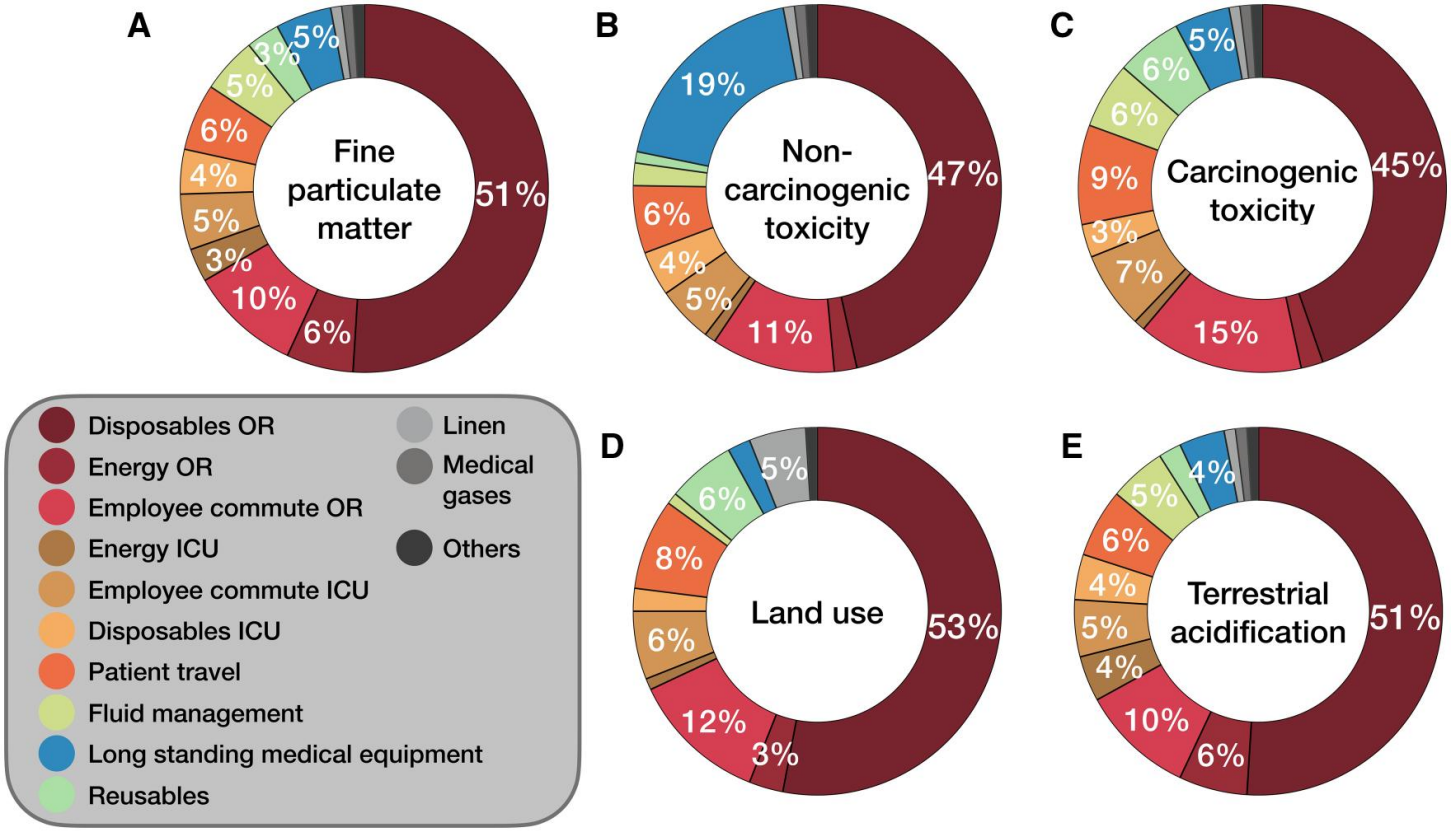
Contribution analysis of a CABG trajectory’s other environmental impacts. Percentages are relative contributions to selected categories, calculated for median values (≤2% not indicated). Disposables, energy use, and employee commute are presented separately for OR and ICU; minor resource groups were combined. Pharmaceuticals were only included for global warming. CABG: coronary artery bypass grafting; ICU: intensive care unit; OR: operating room.

The OR and ICU individually caused 314 [296–333] kg and 77 [68–93] kgCO_2_eq of global warming; patient travel accounted for an additional 23 [19–36] kgCO_2_eq. OR disposables, energy use, and employee commute contributed most: 162 kg (39%), 48 kg (12%), and 36 kgCO_2_eq (9%), respectively (Fig. 5). ICU energy use, employee commute, and disposables made smaller contributions: 31 kg (8%), 18 kg (4%), and 13 kgCO_2_eq (3%), respectively. Fluid management, reusables, medication, long-standing medical equipment, linen, medical gases, and others each contributed ≤5% to global warming. The main hotspots were largely similar to the other reported impact categories (Fig. 6A–E). Long-standing medical equipment, such as ultrasound machine, mainly contributed to non-carcinogenic toxicity (19%) due to the production of printed wiring boards. Further details are reported in Supplementary Material S5.

### Impact of disposables

**Figure 7:**
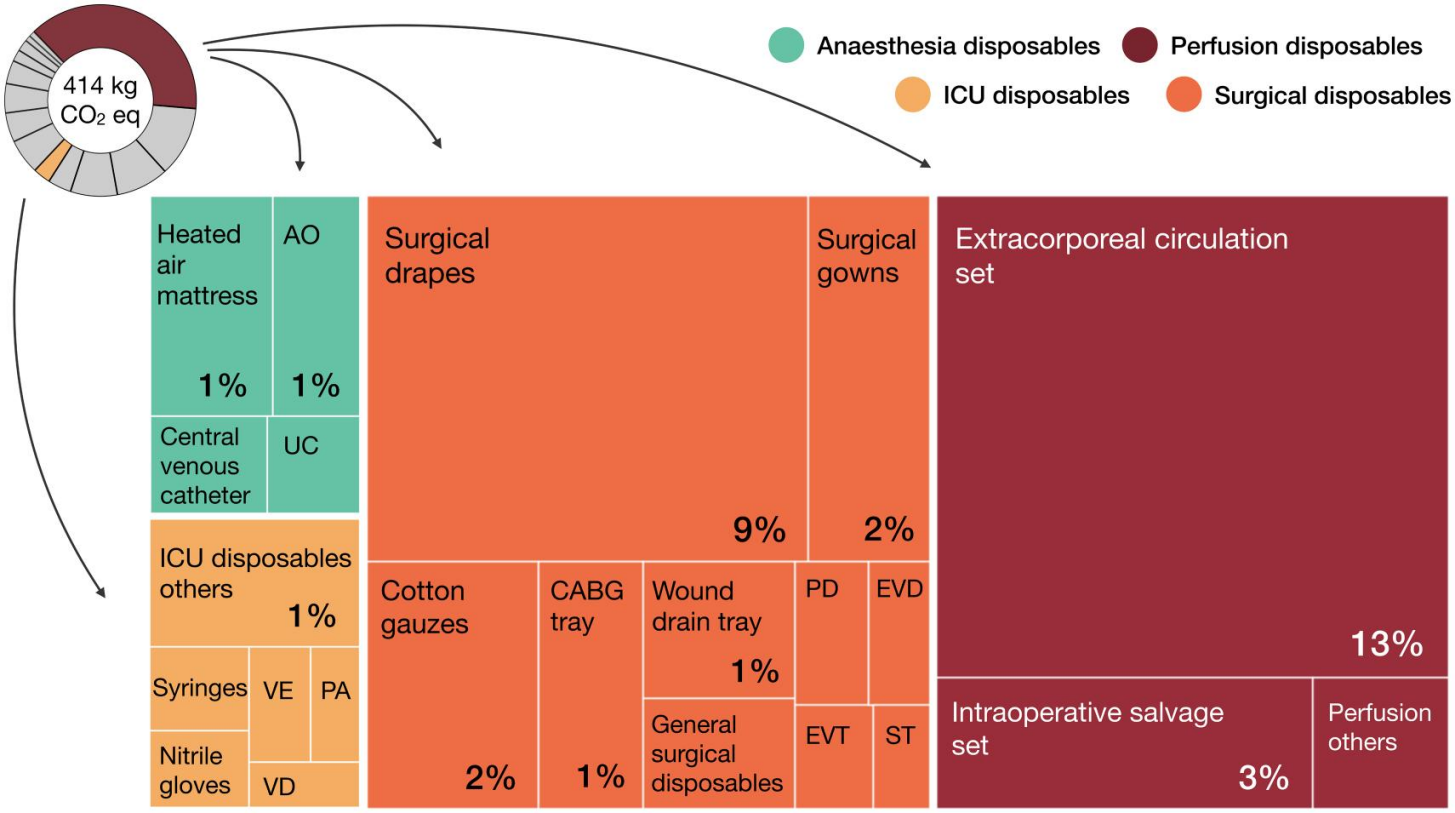
Disposable products’ contribution to a CABG trajectory’s carbon footprint. Percentages are relative contributions to total global warming (414 kgCO_2_eq), calculated for median values (<1% not indicated). AO: anaesthesia others; CABG: coronary artery bypass grafting; EVD: endoscopic vessel-harvesting device; EVT: endoscopic vessel-harvesting tray; ICU: intensive care unit; PA: protective aprons; PD: pleural drain; ST: suture tray; UC: urine collection container; VD: ventilator-related disposables; VE: volumetric exerciser.

Disposables collectively caused 175 kgCO_2_eq (42%) of global warming, with surgical and perfusion disposables as the main contributing subgroups (Fig. 7). The extracorporeal circulation set (55 kgCO_2_eq, 13%), surgical drapes (36 kgCO_2_eq, 9%), intraoperative salvage set (11 kgCO_2_eq, 3%), surgical gowns (10 kgCO_2_eq, 2%), and absorbent cotton gauzes (10 kgCO_2_eq, 2%) caused most global warming. For each of these products, 70–90% of their modelled emissions originated in their production—including high-quality plastic materials. Disposables related to anaesthesia and the ICU contributed less to global warming (3–4% each). Hotspots for impact mitigation were similar to other reported impact categories (Supplementary Material S6). For (non-)carcinogenic toxicity, the incineration of disposables caused most environmental impact.

### Energy use and employee commute

Electricity consumption and natural gas incineration for steam-based air moisturization caused 33 kg (8%) and 4 kgCO_2_eq (1%) of global warming. The HVAC system operated at 68 air changes per hour and used electricity-powered heating and cooling systems. Natural gas and electricity used for washing, disinfecting, and sterilizing of reusables caused most of their impact on global warming (78%, Supplementary Material S6), amounting to 12 kg (3%) and 5 kgCO_2_eq (1%), respectively. Employee commute’s contribution to global warming was caused almost exclusively by car travel (40–50% of OR and ICU nursing staff, others cycled).

### Sensitivity and uncertainty analysis

Choice of an alternative impact assessment method (−2%) and database choices for electricity generation and types of vehicles in use (+5–9%) affected global warming but did not lead to identification of different hotspots than those reported in the manuscript (Supplementary Material S6). Underlying assumptions for disposable incineration (−3%), medication (<1%), reusables (≤1%), and employee commute (1%) neither significantly altered hotspots nor results. Differences for other environmental impacts are reported in Supplementary Material S6. The 95% confidence interval of global warming caused by a single patient CABG trajectory was 359–498 kgCO_2_eq.

### Mitigation scenarios

The largest reductions in global warming could be achieved by exclusively using renewable energy (−66 kgCO_2_eq, −16%) and by employee commute using public transport (−35 kgCO_2_eq, −8%). Replacing disposable surgical drapes and gowns by reusable ones, reducing the HVAC air change rate in the OR, and other possibilities were quantified in Table 1 and detailed in Supplementary Material S6. Other reported impact categories could benefit from the same mitigation scenarios. Additionally, there could be merits to reducing absorptive cotton gauzes (land use) and extended lifetimes of long-standing medical equipment (non-carcinogenic toxicity).

**Table 1:** Scenarios to reduce a CABG trajectory’s environmental impact

Resource category	Scenario	Global warming change kgCO_2_eq (%)
Energy use	Hospital runs entirely on renewable energy, rather than (partially) fossil fuel-powered^a^	−66 (−16%)
Employee commute	Public transportation instead of commute to the hospital by car^a^	−35 (−8%)
Employee commute	All-electric car transportation instead of fossil-fuel-powered cars^a^	−23 (−6%)
Disposables	Reusable instead of disposable surgical drapes^b^	−18 (−4%)
Disposables	Reusable instead of disposable surgical gowns^b^	−7 (−2%)
Energy use	Reduced HVAC air refreshment rate in OR (−16% energy use)^c^	−5 (−1%)
Energy use	Loosen HVAC setpoints for relative air humidity (30% lower limit vs 40%)^c^	−4 (−1%)
Disposables	Reduction of ICU disposables: gloves, protective aprons, and ‘others’ (−30%)^d^	−3 (−1%)

LCA model changes underlying the scenarios are reported in Supplementary Material S6.

a Scenarios based on the studied hospital’s sustainability policies.

b Scenarios based on previous comparisons of disposable/reusable surgical drapes/gowns.

c Scenarios based on calculated HVAC energy use in consultation with in-hospital engineers.

d Scenario based on an assumed 30% reduction of disposables’ weight/quantity.

CABG: coronary artery bypass grafting; HVAC: heating, ventilation, and air conditioning; ICU: intensive care unit; LCA: life cycle assessment; OR: operating room.

## DISCUSSION

A single patient CABG trajectory caused 414 [383–461] kgCO_2_eq of global warming. Other notable environmental impacts were fine particulate matter, (non-)carcinogenic toxicity, land use, and terrestrial acidification. The OR and ICU were, respectively, responsible for 314 [296–333] kg (76% of total) and 77 [68–93] kgCO_2_eq (19%). OR disposables, energy use, and employee commute were key areas for environmental impact mitigation. The extracorporeal circulation set, surgical drapes, intraoperative salvage set, surgical gowns, and absorbent cotton gauzes were the disposables causing most impact. The OR’s HVAC system contributed most to its energy use (77%).

To date, two studies evaluated the carbon footprint of CABG surgery: Grinberg *et al.* and Hubert *et al.* [5, 6]. Similar to our results, disposables were the main group contributing to global warming. However, Grinberg reported 61% fewer (124 kgCO_2_eq) and Hubert 61% more (505 kgCO_2_eq) CO_2_ emissions for surgery. Differences are explained by the use of economical rather than process-based impact quantification, resulting in overestimation, or inclusion of fewer resources in the analysis, resulting in underestimation [23]. These differences underline the importance of standardized methods for environmental impact studies in healthcare [24]. Moreover, Grinberg applied building average values to calculate electricity use and a (French) electricity mix, which largely contains nuclear energy (56 g vs 374 gCO_2_eq/kWh in our setting). Notably, the impact of anaesthesia in our study was lower due to (partial) use of TIVA—which is common practice in the Netherlands and equally effective as volatile anaesthetics [25].

Our findings of 77 kgCO_2_eq for almost 24-h postoperative ICU care broadly align with two prior LCAs of ICU care in the literature. McGain *et al.* found that treating one ICU patient with septic shock was associated with 88 kg CO_2_eq in Australia and 178 kg CO_2_eq in Missouri (USA) [26], whilst Prasad *et al.* similarly found that treating an ICU patient in New York led to 138 kg CO_2_eq emissions [27]. Differences in inclusion criteria (e.g. staff transport was included in this study) make further comparisons imprecise, though the importance of energy and energy sources once again is integral.

Previous environmental impact studies in healthcare illustrated the relevance of considering impact categories other than global warming [2, 3]. In our findings, 53% of harm to human health and 38% to ecosystems was caused by other impact categories: mainly fine particulate matter, (non-)carcinogenic toxicity, land use, and terrestrial acidification. Appreciation of categories other than global warming in environmental impact studies in cardiovascular care, however, is not standard practice—indicating part of the novelty of this study [7]. Whereas the identified hotspots of a CABG trajectory were largely similar across reported impact categories, they were not identical. Especially when proposing alternatives to reduce carbon emissions, trade-offs should be investigated. Bioplastic disposables, for example, incur fewer carbon emissions, yet increase land and water use [28].

Further novelty of this study lies in the hotspot-guided quantification of impact mitigation possibilities. For disposables, we primarily focussed on replacement by reusables for the most contributing products, such as surgical drapes and gowns—based on preceding comparative LCAs (including washing, sterilization, and repair) [29, 30]. Avoiding excessive use of disposable items or removing unused disposables from pre-prepared packs can help to further reduce a procedure’s environmental impact [31]. Critical review of infection prevention measures is required to avoid unnecessary single-use items when strong evidence for clinical superiority is lacking [32, 33]. Extracorporeal circulation use may also offer potential for environmental impact reduction. However, off-pump CABG is technically challenging and has been associated with an increased need for early repeat revascularization and possibly decreased midterm survival [34, 35]. Moreover, it remains to be elucidated how off-pump-associated use of disposables such as stabilizers affect CABG’s environmental impact.

The importance of energy saving was also underlined by our results. Loosening HVAC’s relative humidity setpoints and avoiding excessive air changes are impactful energy-saving measures [36]. Evidence regarding their added value to reduce surgical site infections is equivocal [32, 37]. In addition, energy-saving modes outside of surgical hours are impactful and feasible [38]. Whereas our hospital performs HVAC setbacks when ORs are not in use, we did not include them in this LCA. Last but not least, clinicians can urge hospitals to transition to renewable energy sources (−16% CO_2_ emissions in this study). Differences in other countries may be even larger, if energy is sourced predominantly from coal (Australia) or petroleum and natural gas (USA). Notably, this would also boost the environmental benefit of reusables over disposables, considering the electricity required for washing, disinfecting, and sterilizing [39].

For employee commute, we explored public transport and all-electric car alternatives. Similar contributions (10–11%) of commute to a procedure’s environmental impact were documented in other settings [27]. Considering the number of healthcare professionals required per CABG trajectory, the impact of employee commute may be higher in other hospitals. Had all employees in this study commuted by car for an average distance of 23 km, their carbon emissions would have increased from 55 to 76 kgCO_2_eq. Although no scientific consensus exists regarding the inclusion of employee commute in LCAs, we included it since it offered important impact mitigation possibilities such as the quantified public transport scenario.

Even when combining the largest mitigation possibilities in this study, 75% of a CABG trajectory’s environmental impact remains. Therefore, a transition to sustainable healthcare also requires us to limit CABGs only to those considered to be defined as high value care and advocate for less resource-intensive treatments [8]. Percutaneous coronary intervention (PCI) as a less resource-intensive alternative can be considered [40]. However, randomized clinical trials have shown superior long-term outcomes of CABG over PCI, especially for multivessel disease and diabetes mellitus [41]. In this regard, future challenges include balancing improved environmental sustainability and maintaining optimal medical care.

When converting the driving distance equal to a CABG trajectory’s carbon footprint (2753 km) to petrol costs, these may seem relatively low compared to the cost of a CABG procedure. On the contrary, it should also be noted that medical equipment and procedures are relatively expensive due to, e.g. infection prevention requirements, complex supply chains, and oligopolies [42, 43]. Therefore, a fair and complete inclusion of all relevant environmental impacts in (economical) appraisal of healthcare activities is subject to ongoing research and debate [44, 45].

Several limitations of this study merit emphasis. First, assumptions had to be made mainly regarding pharmaceuticals and material types when data were unavailable, an ongoing difficulty in healthcare LCA research [24]. We used the highest available standards and performed sensitivity analyses to test the robustness of identified hotspots—which remained unaltered. Second, we were unable to include human and ecosystem toxicity of pharmaceuticals in this LCA. Understanding of these impacts may further elucidate the importance of well-considered clinical prescribing. Third, generalizability of results to other hospitals and other types of procedures depends on the quantity/types of materials used, electricity sources, and commuting habits. Despite these limitations, we believe to have generated valuable insights into the impact mitigation possibilities for a CABG trajectory. Moreover, we have captured detailed information regarding the environmental impact of resources commonly used in cardiothoracic procedures and included extensive supplementary information to facilitate (future) comparisons. Further research in other hospitals using a similar standardized and transparent design would strengthen our understanding of CABG’s environmental hotspots and their variability across settings.

## CONCLUSION

The environmental ramifications of a CABG trajectory affect human health and ecosystems, primarily by causing CO_2_ emissions. OR disposables, energy use, and employee commute were key areas for impact mitigation. Promising mitigation possibilities were: avoiding disposable use or replacing them by reusables, loosening HVAC system settings, utilizing renewable energy, and commuting more sustainably. We invite fellow clinicians to incorporate healthcare’s environmental impact into their daily practice and to collaborate in mitigating environmental change to ensure a healthy future.

## Supplementary Material

ezaf054_Supplementary_Data

## Data Availability

Detailed supplementary information facilitates appreciation of the LCA and further use of study findings. LCA data will be uploaded to healthcarelca.com in due time. Further data can be obtained from the corresponding author upon reasonable request within a reasonable timeframe.

## ABBREVIATIONS

CABG: Coronary artery bypass grafting
DALY: Disability-adjusted life years
EuroSCORE: European System for Cardiac Operative Risk Evaluation
HVAC: Heating, ventilation, and air conditioning
ICU: Intensive care unit
LCA: Life cycle assessment
OR: Operating room
PCI: Percutaneous coronary intervention
TIVA: Total intravenous anaesthesia
