# Supplementary material for: Assessing the environmental impact of coronary artery bypass grafting to decrease its footprint
Source: Eur J Cardiothorac Surg. 2025 Feb 17;67(2):ezaf054. doi: 10.1093/ejcts/ezaf054 (PMC11879344; doi:10.1093/ejcts/ezaf054)
Supplement: ezaf054_Supplementary_Data [file ezaf054_supplementary_data.zip › Supplementary_materials_S2-S7_CABG_LCA_EJCTS_[final]_v250221.pdf]

Supplementary material to

Assessing the environmental impact of coronary artery bypass grafting to decrease its footprint

Table of contents

**Supplement S1**	Detailed system boundaries of the CABG trajectory	page 2
**Supplement S2**	Methodological life cycle assessment details	page 2 – 6
**Supplement S3**	Overview of life cycle inventory processes	page 7 – 15
**Supplement S4**	Overview of procedural details and collected primary data	page 16 – 24
**Supplement S5**	Detailed environmental impact assessment results	page 25 – 26
**Supplement S6**	Contribution analysis of environmental impacts, sensitivity analyses, and mitigation scenarios	page 27 – 43
**Supplement S7**	Acknowledgements	page 44

Supplement S1 - Detailed system boundaries of the CABG trajectory

Figure provided separately.

Legend: Overview of resources and their corresponding life cycle stages required for a CABG trajectory (orange square) included in the LCA (within the striped line) and not included (outside of striped line). Legend: CABG = coronary artery bypass grafting; ICU = intensive care unit; HVAC = heating, ventilation, and air conditioning system.

Supplement S2 - Methodological life cycle assessment details

Life Cycle Assessment – introduction of its methodology
A process-based LCA was conducted, consisting of four stages: 1) goal and scope definition by assessing the patient trajectory and translating it to the system boundaries of the material and energy flows and functional unit; 2) inventory analysis describing the environmental inputs and outputs of the material and energy flows of the selected functional unit; 3) impact assessment, which translate the inventory data into environmental impact categories (e.g. global warming (GW), fine particulate matter formation, and land use) and their relation to the environment; and 4) interpretation by critically reviewing all steps of the environmental impact including sensitivity, uncertainty and presentation of the data. Typically, LCA research is an iterative process where different stages are revisited several times to arrive at the final goal and scope definition and corresponding impact assessment. Prior to interpretation of the impact assessment, a completeness and consistency check are performed to verify that enough information has been included to satisfy the goal and scope of the research and to ensure that all inputs in the life cycle inventory (LCI) have been modeled in a consistent way (Laurent et al, 2014).

Life Cycle Assessment – this study
The goal of this LCA was to identify key areas for environmental impact mitigation (hotspots) of a CABG trajectory for a clinical audience. We quantified environmental impacts based on emissions, extractions, processes, or materials associated with the entire life cycle, from raw material extraction to disposal or recycling (‘cradle-to-grave’). ReCiPe 2016 v1.1 (H) was chosen for environmental impact assessment: a widely used methodology to quantify how the subject of study affects 18 different environmental impact categories (the ‘midpoint’ level) and how in turn these contribute to human health damage and ecosystem damage (the ‘endpoint’ level). The endpoint resource scarcity was not considered. For reporting in the manuscript, we chose to focus on the environmental impacts caused by a CABG trajectory that individually contributed most (≥ 10% individually) to damage to human health and ecosystems. This approach for impact category selection was suggested in previous methodological LCA papers. (van Hoof et al, 2013; van Zelm et al, *in press*) Quantification of the environmental impact and analysis of hotspots was mainly performed at the midpoint level for the selected environmental impact categories, such as global warming - the ‘carbon footprint’.

The functional unit of this LCA, which indicates the service or subject to be investigated, was defined as the trajectory of an individual patient undergoing CABG surgery, from OR admission until ICU discharge. (22) Elements considered within the system boundary of this LCA were: disposables, reusables, energy usage, employee commute, patient travel, pharmaceuticals, lab tests, fluid management, linen, medical gases, medical devices, washing

and sterilisation, and waste disposal and treatment. Production of chemicals for cleaning, reagents used for lab tests, and existing hospital infrastructure were excluded based on limited data availability and because they were assumed to have a low contribution to the total impact after allocation to a single procedure. (McAlister et al, 2021; Thiel et al, 2017) Neither were preoperative diagnostics, emergency room visits, stay in the hospital ward, and postoperative follow-up included, considering the feasibility of the amount and quality of data collection by the research team. For all inventory processes, an 'allocation, cut-off by classification' model was used, meaning that the environmental impacts of waste materials are the responsibility of the producer and that valuable by-products of waste treatment become available burden-free (Ecoinvent, 2022).

Study setting

The Radboud University Medical Centre is a tertiary university hospital in Nijmegen, the Netherlands. The hospital has 20 ORs and a dedicated department for thorax surgery, performing 229-341 CABG surgeries annually over the period 2017-2021. Performed surgeries include both patients registered directly at the hospital as well as referred patients from surrounding peripheral hospitals. The majority of CABG surgeries (96.8-99.2%) are performed 'on-pump', using temporary extracorporeal circulation (ECC) and blood cardioplegia whilst bypass grafts are constructed. Being a university training hospital, cardiothoracic surgeons performing the surgery are always accompanied by a surgeon in training. Other staff present during the procedure, such as anesthetists, perfusionists, and surgery assistants, are also frequently joined by a healthcare professional in training. The total number of staff members present in the OR, however, never exceeds the amount of 10.

Data collection

Individual patient trajectories of CABG surgery in the OR and ICU were observed by three dedicated researchers, trained and supervised by an experienced researcher. All products and equipment that were being used during the care process of the patient were registered and, where possible, directly quantified. Protocols, standard care sets, and fixed amounts of materials used, such as a pre-defined 'CABG tray' of disposables and the standardized post-operative ICU room preparation, were identified prior to observations to facilitate the process of input identification. Usage of items in the standardized care sets was verified through observation or consultation of the staff members present during the relevant care activity for every CABG patient. In addition, perfusionists, anesthesiology assistants, and surgical assistants were requested to write down any variable items used during the CABG surgery on standardized sheets and verbally consulted at the end of the surgery as double verification. Information regarding the type and duration of surgery, types and quantity of medication used, and volumes of medical gasses administered were obtained from the hospitals' electronic patient management system. Every surgery, all staff members present were asked for their means of transportation to the hospital that day and the distance covered. Data were extrapolated for ICU staff, considering the large number of healthcare professionals working in the department and shift-based working, which made staff hard to reach. All identified inputs and quantities were combined into one large Excel sheet (Microsoft™, Seattle, WA), providing an exhaustive list of all products and equipment needed. Categories were developed to facilitate grouping of LCI inputs.

Directly after every surgery, all surgery-related waste was collected and transported to a designated waste audit room. Collected waste included all related waste from the separate OR-preparation room and all waste bags and sharps containers within the OR. Packaging waste of pre-op preparations by the perfusionists and anesthesiology assistants, directly

related to the individual CABG surgery yet prepared in different rooms in the OR-building, were collected only once prior to a surgery given their standardized amounts. Quantity and completeness of these pre-operative preparations was verified with multiple staff members. Similarly, given standardized deconstruction and disposal of the ECC-set, this input was only observed and measured once. Sharps bins were collected separately for every procedure, its products safely counted through inspection, and weights obtained by weighing unused equivalents. All other surgical waste was qualified and quantified for every surgery by one or two dedicated researchers. A similar waste audit process was executed for patients after their ICU stay. Waste collection on the ICU took place in designated bins inside the patient room and in a separate small bin outside of the room at the workstation where medication was prepared. Researchers gave frequent presentations to nursing and supporting staff of the ICU department to explain the purpose and methods of the study and to answer any questions. When an included patient was in the ICU, one of the researchers would attend every morning report to make sure that all staff was aware of the waste collection procedure. Products that were used in the OR but only discarded in the ICU, were included in ICU waste audits but allocated to the OR in the data analysis.

Weight, qualitative and quantitative material composition, packaging information, and LCI data of products, such as production, disposal and transportation processes, were obtained in a hierarchical approach: 1) consultation of manufacturers, 2) direct obtainment of information through weighing and available product descriptions; 3) using publicly available data on producer websites to obtain information, and using readily available information in the Ecoinvent database (version 3.9, Switzerland); 4) usage of 'proxy' products for weight and material composition in case similar products have successfully been identified; 5) basing weight and composition on general material characteristics and literature regarding products used in hospitals; 6) applying expert judgement as an assumption for missing information. An overview of data collection per product and process can be found in Supplement B, and can be obtained in more detail from the authors upon request.

Unpacked yet unused medical products were obtained for assessment from healthcare professional training rooms or from observed surgeries or ICU stays where possible. Considering costs and unwanted waste production, no unused medical products were unwrapped and rather weighed inside original packaging, if necessary as entire sets. Products only available inside the OR, such as the reusable equipment sets and disposables inside their original packaging, were weighed using an electronic scale (Kern FFN™) with a precision of 1g, calibrated prior to usage. Products available outside the OR and ICU, such as individual nitrile gloves, were weighed using the AY612 electronic scale (Sartorius™) with a precision of 0.01g, calibrated prior to usage. Large products exceeding the weight limit of this electronic scale, such as the ECC-set, were weighed using a commonly available 'suitcase scale' (ANWB™, the Netherlands) with a precision of 0.01kg. Shipping distances were calculated using a freely available online calculator (searates.com) and road transportation distances were calculated using the navigation function of Google Maps (Google™, Seattle, WA). All travel distances were rounded off to entire kilometers.

The production of capital goods (e.g. OR infrastructure, screens) and the hospital building were outside the system boundary. These products and systems are used for multiple years, a multitude of procedures, and different functional units and therefore have a negligible impact related to the single CABG surgery and ICU stay. Furthermore, the color dye in plastics and paper products and packaging were outside the boundaries due to lack of data. Most similar studies excluded the sterilization of disposable instruments before use as the environmental

impacts are negligible because there are thousands of instruments sterilized at the same time. Still, we included sterilization with ethylene oxide for each disposable. In addition, we did not include the necessary reagents (chemicals) to perform lab tests, considering their small impact compared to the material required to transport the samples (McAlister, 2021). Idle time of machines (such as sterilization units) was not included in this study as the researchers were unable to directly measure energy usage of these devices. A recent proxy process of washer, disinfector, and sterilization units from the UK was used (Rizan, 2022), which did not include idle time in its calculation. Lastly, we excluded the environmental impacts of the surgical teams washing their hands before entering the OR considering the extensive time investment required to monitor this.

For energy use, a similar hierarchical approach was taken as for products – as described previously. As we were not allowed to measure energy consumption of devices in use (to prevent an unwanted scenario where the measurement would disturb the functioning of the device, which might affect the patient), we based power consumption on available data. For the HVAC system, however, we used the hospital engineers' building monitoring system and registration of energy consumption – which allowed for measurement of HVAC components either for specific rooms or for specific areas within the building. For the heating/cooling and air moisturization requirements, we used measurements for an entire calendar year to compute averages. This included all four seasons. Again, details are provided in Supplement B.

Data regarding employee commute were collected in two different ways. For the OR staff, one researcher conducted a brief commute questionnaire with all staff present during every CABG procedure to register travel distance and means of travel. Based on these findings, we calculated a median + IQR for impact assessment. For the ICU staff, we conducted an online commute survey to register their travel distance, means of travel, and the extent to which their travel patterns differed based on night shifts and day shifts. The survey was distributed via the internal team site. Considering the large number of ICU staff, we considered this approach to lead to a more accurate representation of commuting behavior.

Data analysis

Primary analysis of data was conducted using descriptive statistics in the input overview of the Excel sheet. Median values and interquartile ranges were automatically computed for the used quantities of all inputs, omitting missing variables where applicable. All inputs, their corresponding primary and secondary data, and assumptions were thereafter manually modeled into a full life cycle inventory in SimaPro version 9.5.0.1 LCA-software (PRé Sustainability™, Amersfoort, the Netherlands). Where necessary, combined sets of multiple products were modeled as a single input. Ecoinvent version 3.9 (Zurich, Switzerland) market processes were used as background database, unless more detailed information was available to tailor background data to more accurately represent current electricity generation standards or types of vehicles in use in the Netherlands. For all inputs, with the exception of medication, the following processes were modeled: 1) inputs from technosphere assigned as market processes on global, national or regional level; 2) production processes on global, national or regional level; 3) transport market processes to and from the hospital; 4) sterilization of materials where applicable; 5) outputs to technosphere based on material type and method of incineration or empty processes for recycling, in line with the cut-off by classification approach. For reusable items, production and disposal were divided by the amount of times the product was used. Due to limited data availability, pharmaceuticals were only considered for their global warming potential caused by active pharmaceutical ingredient production and packaging. (McGain et al 2021; Myo et al 2021) CO_2 eq-emissions per gram of drug were

used from previous studies or, if no study of the pharmaceutical was available, calculated as an average emission per gram of drug based on the 20 most common OR pharmaceuticals. (Parvatker et al, 2019) Further details of selected processes are provided in Supplement B.

Environmental impact assessment (LCIA) and calculation of environmental impact scores were performed using the ReCiPe 2016 method version 1.1 (RIVM, 2016). ReCiPe is an extensive environmental and epidemiological model which is able to compute the harm caused to human and planetary health by the investigated process. Impact calculation is realized through aggregation and characterization of inventory results. Based on the underlying choice of cultural framework, more weight is assigned to either short term or long-term impacts. For this study, the 'hierarchist' perspective was chosen, balancing both short term and long-term impacts based on scientific consensus and plausibility of impact mechanisms (RIVM, 2016). Prior to impact assessment, LCI inputs were grouped into the pre-defined categories, such as disposables, medication, energy, reusables etc. Environmental impacts of the selected functional unit (CABG surgery + postoperative ICU stay) were chosen to only be presented as 'midpoint' impact categories. An examples of midpoint categories is global warming (GW, kg CO2-equivalent). Reason for doing so is the relative increase of uncertainty of impacts when further aggregation and weighting of results takes place towards 'endpoint' level, such as damage to human health (disability adjusted life years (DALYs)). In addition, for some midpoint environmental impacts the evidence and understanding of the way this environmental impact influences human health or ecosystem functioning is not as well understood. Future research and updates of the impact assessment methodology are likely to improve this understanding and may therefore (partially) alter results.

Uncertainty and sensitivity analysis
Similar to previous LCA-studies (McGain et al, 2010), data uncertainties for categories of inputs were assigned during the LCI compilation using observed variability and a pedigree matrix, converting qualitative process data statements into possible variability distributions (Weidema, 1998; Ciroth et al, 2016). A Monte Carlo analysis was executed to calculate the impacts and obtain 95% CIs to account for variability of LCA-inputs. A Monte Carlo analysis indicates variability of results that could exist, should the study be executed multiple times, performing a large number (≥1,000) of statistical runs. Additionally, several sensitivity analyses were performed to test the robustness of findings based on analysis method selection, data choices for the main hotspots (commute and energy use), and assumptions included in the main hotspots (commute and disposables) and minor categories (medication and reusables). Alternative assumptions were based on existing studies or background data, such as spillage of medication in OR and ICU, to allow for alternatives that were based on real world scenarios.

Mitigation scenarios
As pointed out in the main manuscript, we performed a hotspot-guided quantification of environmental impact mitigation possibilities based on hypothetical scenarios – informed by current policies, existing literature, or expert-informed assumptions. Each scenario was entered into the LCA-modelling (specifically: by creating alternative inputs in the life cycle inventory), after which re-calculation of environmental impacts was performed to determine the potential reduction (percentage) of environmental impact. Details regarding LCA-model changes are provided alongside results in the respective supplementary table (Supplement F).

Supplement S3 – Overview of life cycle inventory processes

**Process category**	**Subcategory**	**Data collection**	**Data modelling in LCI**
Disposables OR	Perfusion disposables - extracorporeal circulation (ECC) set	Type and quantity based on observations in the OR and the perfusionist room where machines are assembled & disassembled; interviews with staff (perfusionists); obtained complete disposable ECC-set previously used for training purposes to examine and weight (larger) components; materials/compostion based on interviews with staff and researcher assumptions	Own modelling using ecoinvent market processes. Since we did not disassemble the entire set for individual components (such as the internal filters and the metal coil), we made assumptions regarding the exact weights of components. Except for the plastic case, the entire set is incinerated as 'hazardous' medical waste.
	Perfusion disposables - intraoperative salvage (IOS) set	Type and quanitity based on observations in the OR and the perfusionist room where machines are assembled & disassembled; interviews with staff (perfusionists) and weighed the entire set including case in the perfusionist room; materials/compostion based on interviews with staff and researcher assumptions	Own modelling using ecoinvent market processes. Since we did not have an unused set at our disposal, we made estimations of the weights of different materials. Except for the plastic case, the entire set is incinerated as 'hazardous' medical waste.
	Perfusion disposables (others)	Type and quantity based on observations in the OR, waste audits, and surgical protocols. Part of the disposables that appeared relatively frequently (e.g. syringes) were counted individually and disassembled/weighed for their components; other disposables (e.g. aortic punch) were weighed including their packaging; for other disposables that were unavailable for weighing, the weights and materials were assumed based on observations (e.g. suction tubes, mosquito clamps, rubber slings)	Own modelling using ecoinvent market processes. Since we did not have unused products/versions of all components available, we made estimations of the weights of different materials and used proxies for similar products that had been studied/weighed in other "trays" or individual components in this study.
	Surgical disposables - patient, device, table covers (Surgical drapes)	Type and quantity based on observations in the OR, waste audits, and document analysis (surgical protocol); weighed unused surgical drapes and estimated weight of other surgical drapes based on relative size differences; materials based on information received from manufacturer; estimated relative composition of materials based on manufacturer information regarding thickness	Own modelling using ecoinvent market processes. Drapes were originally modelled as part of the disposables trays (e.g. "CABG tray"), since they were part of that tray in the inventory list. For further analysis, a separate group for only the surgical drapes was created.
	Surgical disposables - surgical gowns	Type and quantity based on observations in the OR, waste audits, and document analysis (surgical protocol); weighed unused surgical gowns; materials based on information received from manufacturer; estimated relative composition of materials based on manufacturer information regarding thickness	Own modelling using ecoinvent market processes.
	Surgical disposables - cotton gauzes	Type and quantity based on observations in the OR, waste audits, and document analysis (surgical protocol); weighed complete 10p package of cotton gauzes and estimated individual weight; materials based on online information regarding abdominal XRD gauzes for absorptive use, assumed weight of barium thread	Own modelling using ecoinvent market processes. Cotton gauzes were originally modelled as part of the disposables trays (e.g. "CABG tray"), since they were part of that tray in the inventory list. For further analysis, a separate group for only the cotton gauzes was created.

	Surgical disposables - "CABG tray"	Type, individual contents, and quantity based on document analysis (surgical protocol) and observation of material preparation in the preoperative material preparation room. Since we were not able to obtain a complete, unused disposable set for further analysis, we weighed individual components when available (e.g. syringes and needle box) and estimated weights of other included components (e.g. extra tubes connected between patient and ECC-machine; or cellulose OR towels)	Own modelling using ecoinvent market processes. Whereas the 'tray' consists of a multitude of disposable objectes, the choice was made to create one input process in the model - as the entire tray appears as one in surgical protocols. Further details of contents can be found in the corresponding process tab. During contribution analysis, subgroups of disposables that appeared in multiple trays and contributed significantly to the overal environmental impact (.e.g. surgical drapes), were also modelled in separate processes.
	Surgical disposables - "Corridor A tray" a.k.a. "Wound drain tray"	Type, individual contents, and quantity based on document analysis (surgical protocol) and observation of material preparation in the preoperative material preparation room. Since we were not able to obtain a complete, unused disposable set for further analysis, we weighed individual components when available (e.g. smoke pencil) and estimated weights of other included components (e.g. silicone wound drain)	Own modelling using ecoinvent market processes. Whereas the 'tray' consists of a multitude of disposable objectes, the choice was made to create one input process in the model - as the entire tray appears as one in surgical protocols. Further details of contents can be found in the corresponding process tab.
	Surgical disposables - general surgical disposables	Type and quantity based on observations in the OR, waste audits, and surgical protocols. Disposables were counted individually and disassembled/weighed for their components when possible (e.g. gloves, theatre caps), materials were assumed when no product information was available; other disposables that were unavailable for weighing, the weights and materials were assumed based on observations (e.g. suction tube). Usage of (hand) desinfectant based on previous study and assumed amount for patient skin desinfectant.	Largely own modelling based on ecoinvent market processes. Products were modelled individually and combined into one overarching process. Nitrile gloves included as elaborated on below (ICU disposables). Surgical gloves based on Weisz et al (2020). Face masks based on LCA by Lun Lee et al (2021) . Desinfectant based on handwashing LCA by Duane et al (2022), wherein we assumed patient desinfectant to be the same substance as hand desinfectant - in the absence of product information related to the patient skin desinfectant.
	Surgical disposables - "Endoscopic vessel tray"	Type, individual contents, and quantity based on document analysis (surgical protocol) and observation of material preparation in the preoperative material preparation room. Since we were not able to obtain a complete, unused disposable set for further analysis, we weighed individual components when available (e.g. filter or the abdominal insufflation tube) and estimated weights of other included components (e.g. plastic drape of endoscopic camera cable)	Own modelling using ecoinvent market processes. Whereas the 'tray' consists of a multitude of disposable objectes, the choice was made to create one input process in the model - as the entire tray appears as one in surgical protocols. Further details of contents can be found in the corresponding process tab.
	Surgical disposables - "Disposable kidney tray" a.k.a. "Suture tray"	Type, individual contents, and quantity based on document analysis (surgical protocol) and observation of material preparation in the preoperative material preparation room. Since we were not able to obtain a complete, unused disposable set for further analysis, we weighed individual components when available (e.g. sutures) and estimated weights of other included components (e.g. ligation clips).	Own modelling using ecoinvent market processes. Especially for the sutures, proxies had to be used since the materials themselves are not available in ecoinvent (or: are unknown). Whereas the 'tray' consists of a multitude of disposable objectes, the choice was made to create one input process in the model - as the entire tray appears as one in surgical protocols. Further details of contents can be found in the corresponding process tab.
	Surgical disposables - pleural drainage system	Type and quantity based on observations in OR and document analysis (surgical protocol). Since we were not able to obtain a complete, unused drain reservoir, we estimated the weight and material types of the reservoir based on observations and comparison with other types of plastic in this study (e.g. the hard plastic reservoir resembled the hard plastic of the ECC-set).	Own modelling using ecoinvent market processes.

	Surgical disposables - endoscopic vessel harvesting (EVH) device	Type and quantity based on observations in the OR, waste audits, and surgical protocols. The EVH devices were counted individually and were once disassembled/weighed for their components, materials were assumed when no product information was available.	Own modelling using ecoinvent market processes.
	Anaesthesia disposables - heated air mattress	Type and quantity based on observations in the OR, waste audits, and document analysis (surgical protocol); weighed the product; materials based on information received from manufacturer; estimated relative composition of materials based on manufacturer information regarding thickness	Own modellig using ecoinvent market processes.
	Anaesthesia disposables - central venous catheter insertion set	Type and quantity based on observations in the OR, interviews with staff, and document analysis (surgical protocol); weighed a similar complete set of the product (no used separate materials available for weighing); materials assumed or based on available information on manufacturer website; estimated relative composition of materials based on size of objects	Own modelling using ecoinvent market processes.
	Anaesthesia disposables - urine collection container	Type and quantity based on observations in OR, ICU, and document analysis (surgical protocol). We estimated the weight and material types of the reservoir based on measurements of the products inside their packaging (e.g. the hard plastic reservoir) or on observations if no complete set was available (e.g. the catheter insertion set). Estimations were made for material type and composition, based on comparison with other types of plastic in this study (e.g. the hard plastic reservoir resembled the hard plastic of the ECC-set).	Own modelling using ecoinvent market processes.
	Anaesthesia disposables (others)	Type, individual contents, and quantity based on observations in the OR, waste audits, interviews with staff, and electronic hospital records. Disposables were counted individually and disassembled/weighed for their components when possible (e.g. syringes, breathing circuits, forehead electrodes), materials were assumed when no product information was available; other disposables that were unavailable for weighing outside of their packaging, the weights and materials were assumed based on observations (e.g. arterial catheter).	Own modelling based on ecoinvent market processes. Products were modelled individually and combined into one overarching process. Overlap exists with individual processes (such as urine collection container) which was excluded as a separate process during the contribution analysis. The anesthetic breathing circuit was assumed to be used for 10 surgeries/patients and was only partially allocated.
	Medical waste incineration	Categorised which disposables would be incinerated via high temperature medical waste incineration, based on observations and waste audits.	Modelling based on previous work by Bodegraven et al (2023) for the Dutch Institute for Public Health and the Environment's (RIVM) report on facemask use during the COVID19-pandemic. Includes (an allocated share of) the polypropylene bin used to collected 'hazardous' medical waste and a specific incineration process including direct emissions to the environment, based on previous studies of Zhao et al (2009, LCA of medical waste incineration) and Leeuw & Koelemeijer (2022, decarbonisation options for the Dutch waste incineration industry).<br><br>Modelling not provided in this supplement, but can be obtained via contact with the original authors (Martijn van Bodegraven, Dutch Institute for Public Health and the Environment)

Disposables ICU	ICU disposables	Type and quantity based on observations in the ICU, waste audits, document analysis of room preparation inventories, and interviews with staff. Disposables were weighed and assessed for material composition individually when available (e.g. nitrile gloves, protective aprons, syringes), based on previous research when available (e.g. face mask or absorbent underpads), or based on assumptions + observations when necessary (e.g. volumetric exerciser).	Own modelling using ecoinvent market processes. The majority of disposables (especially those ones assumed to have the largest environmental impact individually) were modelled separately (e.g. the forced-air warming blanket, the absorbent underpads, nitrile gloves [Jamal et al 2021]). For some (smaller) disposables, previously modelled disposables that were considered to be similar in term of materials or types of usage (e.g. scalpel and stitchcutter) were used as proxies.<br><br>Groups of diposables (e.g. syringes, ventilator-relate disposables) were created that had a notable (≥ 1%) impact on the total impact of the CABG trajectory. The diverse, remaining majority of disposables was included in a larger group of "others", such as: in-ear thermometer covers, disposable coffee cups, glucose test strips, individual band aids/bandages. The process is included in the "ICU disposables" tab.
Energy OR	OR energy - heating, ventilation, air conditioning (HVAC)	Quantity of HVAC energy use based on measured annual average electricity consumption of HVAC for the OR. Fresh outside air is introduced from a central entry supplying 5 ORs and a clean room at the same time (energy consumption therefore allocated), recirculation of air happens in every OR individually (directly measured). Calculated required natural gas for air moisturisation based on amount of outside air that needs to be moisturised, accounting for piping losses (15%) and incineration efficiency (85%) of steam kettles - this happens in a central boiler room for the entire hospital. Air is heated and cooled using a system of coils (electricity-powered). End-of-pipe heating just before air entry in ORs was not considered, due to inability to measure variations based on staff air temperature settings. All data were collected in close collaboration with the specialised hospital HVAC engineer.<br><br>Service hours based on reporting of surgery duration in electronic hospital records and includes 2h perioperative time (during which the OR HVAC is already on). Require amounts of demiwater for steam generation and water for cooling were adopted from annual data (m3 water/m3 air) of another Dutch hospital.<br><br>For technical reference: The OR (48m2 x 3m) in which surgeries are performed has a unidirectional air flow system (UDAF) with a central 'fresh outside air' outlet (2,000 m3/h) and peripheral recirculated air outlets at 1.5K higher temperature (7,775 m3/h), resulting in 68 air changes per hour through HEPA filters. This is classified as an 'ultra clean OR' following Dutch professional standards.	The electricity mix tailored to the Dutch market in 2021 "Elektriciteit gemiddeld - NL 2021" is a market process (S) that was created by CE Delft (Delft, the Netherlands) in 2023-2024, commissioned by the Dutch Government and based on energy generation data as collected by the Dutch National Institute for Statistics (CBS) and National Planning Bureau for the Environment (PBL). Documentation related to its creation can be found via this website (only available in Dutch): https://cedelft.eu/wp-content/uploads/sites/2/2024/02/CE_Delft_230126_Ketenemissies_elektriciteit_Def.pdf<br><br>Modelling based on electricity use per hour and multiplied by the duration of the surgery. Since annual data are used, the calculation contains both heating and cooling of fresh outside air (which does not take place at the same time in reality). Energy consumption of each component (e.g. recirculation ventilator) modelled separately. The choice was made to add the required 'primary energy' for heating or cooling of air as direct electricity consumption. In reality, energy use for the hospital under study would have been lower, since heat pumps and thermal storage are used, which would lead to efficiencies (COP) of 3-4. However, since this was not considered as 'usual' for the average hospital, we chose not to include it. Moreover, required amounts of primary energy could be used to calculate scenarios of energy use for heat networs using natural gas incineration (or other alternatives).

	OR energy - devices	Quantity of electricity use related to equipment based on observations of devices (in use) in the OR. Information regarding energy consumption of different devices was based on previous publications (Drinhaus et al 2023), product manuals available online, and an overview of energy consumption measurements of similar equipment in different ICUs in the Netherlands ("Werkblad Groene IC", available online soon: www.degroeneic.nl).	Explanation of electricity mix as stated in "OR energy - HVAC"<br><br>Modelling based on (estimated) electricity use per hour when active ("during surgery") and in standby when no surgery is performed ("presurgery"), multiplied by the duration of the surgery plus two hours of perioperative time (not all devices are swtiched on during periopreative time, but the anesthesia carestation and the computers are turned on).
	OR energy - light	Estimation of hourly usage for lighting by in-house energy specialists and engineers, based on technical data for power consumption of OR lighting (0.54kWh = 54W * 10 lamps)	Explanation of electricity mix as stated in "OR energy - HVAC"<br><br>Modelling based on (estimated) electricity use per hour and multiplied by the duration of the surgery plus two hours of perioperative time.
Energy ICU	Total ICU energy consumpt ion	Quantity of HVAC energy use based on measured annual average electricity consumption of HVAC for the whole ICU, allocated to a single unit. Calculated required natural gas for air moisturisation based on amount of outside air that needs to be moisturised, accounting for piping losses (15%) and incineration efficiency (85%) of steam kettles.<br><br>For technical reference: we allocated 20 m2 surface area for the patient room and an additional 20 m2 of general area. Room height 3m. Air changes: 6 per hour.<br><br>Quantity of electricity use related to lighting and equipment based on observations of devices (in use) and lighting in the ICU, both in general/shared areas and inside the patient room. Information regarding energy consumption of different devices was based on previous publications (Drinhaus et al 2023), product manuals available online, and an overview of energy consumption measurements in different ICUs in the Netherlands ("Werkblad Groene IC", available online soon: www.degroeneic.nl).	Explanation of electricity mix as stated in "OR energy - HVAC". Modelling of energy use of HVAC similar to explanation of "OR energy - HVAC".<br><br>Modelling based on electricity use per hour (in Watt or kWatt) and multiplied by the number of hours the patient was admitted to the ICU or received the respective therapy (e.g. energy use of the ventilator was only considered for the duration that the patient received invasive ventilation). Where necessary, information regarding duration of use (e.g. forced heating blanket) was assumed.<br><br>For the general/shared area, electricity consumption of devices was evenly allocated based on observed bed occupancy in the respectve unit. Where necessary, assumptions were made when e.g. lights are turned on or switched off.

Employee commute	Home-work commute of OR (and ICU) employees	Type and quantity based on direct interviews with staff present in OR: registered travel distance to the OR and the means of travel. In addition, conducted a commuting survey (means and distance) among ICU staff (n=121).	The transportation processes tailored to the Dutch market for transportation of goods (Goederenvervoer STREAM 2020; CE Delft, 2021) and commute of persons (Personenvervoer STREAM 2022; CE Delft, 2023) were used as market processes (S/U), adjusted from the standard cut-off by classification market processes in ecoinvent v3.9.1 for the same fuel types and vehicle types. The adjusted processes were previously commissioned by the Dutch Government. Adjustments are made for the processes that represent the production of fuels, such as gasoline (i.e. Well-To-Tank emissions), e.g. gasoline currently contains 10% biofuels, rather than 5% biofuels, resulting in a lower requirement of crude oil and higher requirement of land use for production of biofuels. And adjustments are made for the processes that represent the combustion of fuels and driving of vehicles (i.e. Tank-To-Wheel emissions), e.g. the amount of PM2.5 that is emitted during the process of driving a car, based on direct measurements of Dutch vehicles. The report of the 'Personenvervoer 2022' can be found here (in Dutch): https://ce.nl/wp-content/uploads/2023/02/CE_Delft_210506_STREAM_Personenvervoer_2022_DEF.pdf<br><br>Effectively, this results in: 1) replacement of the CO2eq, NOx, SO2, NMVOC, and PM2.5 emissions documented in ecoinvent processes by the vehicle measurements conducted in the Netherlands; 2) for commute of persons the reporting unit of cars and motorbikes was adjusted based on the average occupation of vehicles ('km' to 'personkm'), such as a factor 1.31 for the average car. For further details/reporting, kindly consult the authors.<br><br>Own modelling of travel distance for the entire surgical team, resulting in the total return distance per means of travel, divided by 2 (since a 50% allocation is chosen considering that 2 cardiac surgeries are performed by the same team per day). For the ICU team, we allocated responsible staff based on 24h staffing schedules for our ICU, evenly divided by the (observed) average bed occupation in the ICU
Patient travel	Travel to the hospital for patient	Distance calculated based on electronic hospital records (using ZIP codes and travel distance to the hospital)	Explanation of transportation process as explained for "Employee commute"<br><br>Own modelling of (return) travel distance for a single person, assuming all patients travel by car (most likely, considering that they are visiting for the purpose of a surgery); selected 'average car mix', consisting of petrol, diesel, and electric cars.
Fluid management	i.v. fluid bags	Type and quantity based on observations in OR and ICU, combined with electronic hospital records	Own modelling using ecoinvent market processes. All i.v. fluids were modelled based on the production process of a 500mL crystalloid (NaCl0.9%) - assuming that other types of fluids had a similar impact for production as the NaCl. Plastic of fluid bags was assumed to consist of 3 types of plastic (in equal division) based on personal communication. For the impact of blood transfusion (packed cells), only kg CO2eq were considered based on a previous publication (Hibbs et al 2024).

Medica tion	Pharmac euticals	Type and quantity based on pharmaceuticals administration registered in electronic hospital records<br><br>Includes pharmaceuticals administered perioperatively and in the ICU, excluding medication that the patient already used prior to surgery (e.g. metformine)	Modelling based on several previous publications, directly entered as kg CO2eq per gram of active pharmaceutical ingredient (API). Includes morphine (McAlister et al 2016), the 20 most frequently used OR drugs (Parvatker et al 2019), paracetamol (Davies et al 2024), sevoflurane (Sherman et al 2012), and albumine 20% (EPD available online, 2019). For the pharmaceuticals that were not included in the aforementioned publications, an average emission (kg CO2eq) per gram of drug was calculated base on the Parvatker (2019) study.<br><br>For all pharmaceuticals we aso included an emission related to the packaging, either as blister packaging or as glass/plastic container, based on the Myo et al (2021) study.
Device s OR + ICU	All devices	Type and quantity based on observations of devices being used in the OR and ICU and interviews with staff<br><br>Includes surgical, perfusion, anaesthetic, ICU, and imaging related devices, such as a vital parameters monitor or ventilator. Information on material composition for production of devices and number of times used based on confidential information of medical device manufacturer (cannot be shared at this time due to a non-disclosure agreement).	Own modelling using ecoinvent market processes. Since only one of the medical device manufacturers was willing to provide information on the components of the devices, we chose to create a rough proxy process wherein we modelled the devices available and made an estimation how other devices would relate to the ones we modelled. E.g.: a vitals monitor relates directly to the one used in the OR, an ultrasound machine was used as a direct proxy (considering that the one used in the OR is made by a different manufacturer), and the extracorporeal circulation machine was assumed to be 'equal' to the production of an ultrasound machine.<br><br>All devices were assumed to be used 1500 times, based on a 3 year usage duration - which is on the conservative side based on device usage in our hospital (234 working days, 2 surgeries per day, and an additional 100 non-elective procedures outside of office hours).
Reusab les	Surgical trays	Type and quantity based on observations in the OR; weighed reusable trays (including tray) on site; contains trays of surgical steel instruments, including 4 cardiothoracic-tailored sets, a magnetic mat, a sternal saw, and the reusable laryngoscope blades.	Own modelling using ecoinvent market processes. Assumed chromium steel 18/8 for all instruments and wrapping of sterilised trays using polypropylene wraps. Assumed usage of main trays of instruments of 200 times based on conversations with central sterilisation department. Since composition of the magnetic mat was unknown, we assumed material composition and weights as an AlNiCo magnet.<br><br>For the modelling of the laryngoscope (blades), a previous study by Sherman et al (2018) was used to determine weights, composition of e.g. handle including light conducting fibre optic glass, and times used (assumed 400 for the reusable components and 40 for the battery).

	Washing, disinfecting, sterilisation	Washing, disinfection, and sterilisation process based on previous publication(s); consulted sterilisation department of own hospital for comparability	Modelling based on Rizan et al (2022) washing, desinfection, and sterilisation study; adjusted to suit reusables in own hospital. Energy use, tap water, and soap (ecoinvent processes) were taken as total amounts required per cycle of a fully loaded machine, divided by the amount of slots available, and multiplied by 2 for the main reusable trays (considering that the instruments are spread over 2 trays during the washing/desinfecting process to make sure that all surfaces are cleaned). Full loading was assumed, based on conversations with the central sterilisation department. In the sensitiviy analysis we also investigated the impact for the audited occupancy rates of slots as reported by Rizan et al.<br><br>In the sensitivity analysis, the sterilisation process mapped for a vaginal delivery set in another Dutch academic hospital was used for comparison (CE Delft 2021), which was equally adjusted to suit the size of reusable trays and the type of steam in our own hospital. Available here (in Dutch): https://cedelft.eu/wp-content/uploads/sites/2/2022/12/CE_Delft_220162_Eenmalige_of_herbruikbare_partus-_en_hechtsets_Def.pdf
Linen	Scrubs, bed linen, staff clothing	Type and quantity based on observations and waste audits<br><br>Includes bed linen (bed sheet, blanket, pillowcase), patient gown, and towels. Also includes OR scrubs (tops + pants) and hospital wear of employees (white coat, white pants, white top/polo shirt).	Modelling based on Thiel et al (2015) Hysterectomy LCA and own adjustments. Weights and materials of different types of linen (ecoinvent market processes) were taken from the 2015 publication and then adjusted for the number of times the average piece is used based on average data of the external laundry service that washes all linen for the hospital (i.e. 100 times average use for bed linen and 130 times average use for OR scrubs and hospital wear). Thereafter combined with laundering process - which is detailed below.
	Laundering process	Information regarding washing process obtained from external laundry service (Nedlin, the Netherlands) that is contracted by the hospital.<br><br>Since laundry takes place at industrial scale for multiple hospitals and care organisations at the same time, high efficiency of processes can be attained. Optimisation processes to re-use water in laundry processes is in place.	Own modelling using ecoinvent market processes. Since detailed informtion regarding laundry detergents was unavailable, considered regular "Soap {GLO}" and sodium perborate tetrahydrate as components. Applied dilution factor of 0.5 (mixed with deionised water) to convert weight to liquid soap and a 0.75 'sustainability factor' (own assumption), based on information by laundry service that current soap has a lower environmental impact. Model also includes elektricity use and natural gas use for heating or drying and transporation of linen to and from the hospital.
Medical gases	Oxygen, carbon dioxide, compressed air	Quantity based on observations in the OR, electronic hospital records, and manual calculations for ventilator gas usage (O2 and compressed air)<br><br>Includes oxygen, carbon dioxide, and compressed air used in the OR and ICU for respiration and insufflation	Own modelling using ecoinvent market processes for liquid oxygen and carbon dioxide, with added transportation to hospital assuming transportation by lorry with a refrigeration machine (as proxy for specialised truck/lorry). Compressed air only added as market process, since it is produced on site and not transported to the hospital.

Others	Lab tests	Type and quantity based on electronic hospital records and observations in the OR<br><br>Includes arterial blood gas syringe, the blood proteins/hormonal /kidney function tests, the coagulation tests, and the haemocytometry tests	Modelling based on McAlister et al (2021) LCA of hospital pathology testing. Considering that main impacts were reported to depend on material and energy usage, the chemical reagents and tube coatings were assumed to be of minimal impact and exlcuded from analysis (due to their very small weight). Energy usage includes the energy to run one test and does not include the idle time of the machine.<br><br>For every moment that blood tests were performed, similar test that could be performed using the same sample tube (e.g. blood proteins and kidney function) were assumed to be performed using one and the same sample. Glucose POCT diagnostics were modelled using 'blood proteins/hormonal' as a proxy, since no information on bedside glucose testing was available.

NB 1: for all the disposables in the overview above, transportation processes from (assumed) production site to the hospital have been included, almost exclusively via sea containers and lorry transport from the harbour to the hospital.
NB 2: a general compensation of 'missing transport' has been added to the LCA to cover for the transportation of disposables between intermediary retailers (for the total weight of disposables used in the LCA), consisting of 150km lorry transport, 75km train transport, and 75km container ship transport.
NB 3: for all disposables a sterilisation process using ethylene oxide (gas) has been added; this process was based on the ethylene oxide process included in McAlister et al's (2021) LCA of hospital pathology testing.
NB 4: for all disposables, plastic- and metal-specific incineration processes were modelled; if specific plastics were unavailable or if disposables consisted of other materials, the municipal incineration or medical waste incineration (where applicable) were chosen.

Note to reader

Considering the substantial amount of time required to export processes from SimaPro and format them into a readable version in this Supplementary data, we have currently not provided the life cycle inventory for each of the processes and subcategories listed above. To report transparently and to further the field of LCA research in healthcare, the authors plan to upload all relevant processes to the open access healthcarelca.com database in due time. Moreover, a supplementary Excel file containing all exported inventories is available upon reasonable request within a reasonable timeframe.

Supplement S4 – Overview of procedural details and collected primary data

Table D1. Procedural descriptives

Sex	Age	EuroSCORE II	Procedural details *	ECC-duration (minutes)	AoX-duration (minutes)	OR duration (hours)	ICU length of stay (hours)	ICU intubation duration (hours)
M	73	0.8	CABG 2 DA (LIMA-LAD vein-PLCx)	95	56	4.8	31.3	14.5
F	60	0.9	CABG 4 DA (LIMA-LAD, Ao-(vsm)-D-MO-RCA)	126	85	4.6	22.7	5.2
M	62	1.8	CABG 4 DA (Lima-LAD, Ao-VSM-D-MO-RDP)	103	63	4.6	23.4	8.4
F	63	1.0	CABG 3 DA (LIMA-LAD, Ao-(vsm)-PLCx-RCA)	69	42	4.0	24.3	7.7
M	65	1.5	CABG 4 DA (LIMA-LAD, Ao-(vsm)-MO-RPL-RDP)	86	60	4.2	19.7	12.2
M	73	1.9	CABG 4 DA (LIMA-LAD, Ao-(vsm)-AL-MO-PLCx)	87	57	4.8	21.5	13.8
M	74	3.1	CABG 5 DA (LIMA-LAD, Ao-D-MO1-MO2-RDP)	80	54	5.0	19.8	8.0
F	62	1.2	CABG 3 DA (LIMA-LAD, Ao-vein-MO-RDP)	85	36	4.5	69.5	31.9
M	77	1.7	CABG 5 DA (LIMA-LAD, Ao-D-MO-RPL-RDP)	87	59	3.7	20.5	9.2
M	74	0.8	CABG 5 DA (LIMA-LAD, Ao-D-AL-PLcx-RDP)	158	86	5.6	20.0	12.7
F	75	2.0	CABG 4 DA (LIMA-LAD, Ao-(vsm)-D2-MO-RPL)	104	69	4.9	46.9	4.2
M	66	1.6	CABG 3 DA (LIMA-LAD, Ao-(vsm)-D-AL)	77	40	3.9	19.1	4.1
	Median [IQR] age	Median [IQR] EuroSCORE II	Median [IQR] no. anastomoses	Median [IQR] ECC-duration	Median [IQR] AoX-duration	Median [IQR] OR duration	Median [IQR] ICU length of stay	Median [IQR] intubation duration
	70 [63–74]	1.5 [1.0–1.8]	4 [3–4]	87 [84–103]	58 [51–65]	4.6 [4.2–4.9]	22.1 [20.0–26.0]	8.8 [7.1–13.0]

* Please note that endoscopic vessel harvesting was used to obtain grafts for all procedures (not included in the table)

Table D2. Quantities of waste types (kg)

Waste type	Operating Room, Surgical theatre			Intensive Care Unit, Patient room + medication preparation station		
	Median	Q1	Q3	Median	Q1	Q3
Hard plastics	0.37	0.34	0.52	0.15	0.10	0.18
Soft plastics	0.21	0.17	0.42	0.30	0.25	0.44
Paper	0.24	0.13	0.41	0.22	0.14	0.23
Polypropylene sheets (OR only)	0.29	0.22	0.54	n/a	n/a	n/a
Laminates	0.35	0.32	0.43	0.10	0.10	0.14
Tubes & syringes	1.95	1.70	2.26	2.32	1.47	2.63
General waste (other)	8.00	7.63	8.38	1.49	1.29	2.06
Hazardous waste	7.87	7.87	7.87	1.76	1.58	2.63
	Operating Room, Sterile preparation room			Intensive Care Unit, **Total ***		
Plastics (recycled)	2.93	2.86	3.05	6.07	4.97	7.23
Paper (recycled)	3.98	3.59	4.13			
Polypropylene sheets	0.89	0.76	0.95	**Total waste**, Operating Room + Intensive Care Unit		
General waste (other)	1.52	1.43	2.54	35.18	32.09	38.38
	Operating Room, **Total ***					
	29.11	27.12	31.15			

*Please note that the median and quartile total values of the OR and ICU do not equal the sum of the median and quartile values of the different waste types. Reason for this is that for the observed procedures, a single procedure does not necessarily contain the same median/quartile value for all different waste types (but rather a combination of values). To more accurately represent the total amount of waste for the observed procedures, the median and quartile values for the procedures' total waste were calculated.

Table D3. Disposable products used per surgery (in the OR)

Product	Additional details	Median (N)	Q1 (N)	Q3 (N)
Perfusion disposables				
Extracorporeal circulation set	Medtronic custom pack, weighs 6.4kg; containing polycarbonate blood jars (±33%), PVC tubing (±33%), minor components / membranes / foam structures assumed polypropylene and polyurethane, fibre-like structures assumed polyester, and metal coil assumed to be chromium steel.	1	1	1
Intraoperative salvage set	Sorin CellSaver Xtra pack, weighs 1.5kg; containing polycarbonate blood jar (±45%), PVC tubing (±45%), membranes assumed polypropylene, metal components assumed chromium steel.	1	1	1
Perfusion 'others'				
- 1mL syringe		1	2	5
- 3mL syringe		2	0	0
- 5mL syringe		2	0	3
- 10mL syringe		1	1	1
- 20mL syringe		0	0	0
- 50mL syringe		1	0	2
- i.v. spikes		4	4	4
- blunt filling needle		1	1	2
- cannulas (venous/arterial)		2	2	2
- ECC connectors/adapters		3	3	3
- ACT cartridges		3	3	4
- mosquito		1	1	1
- dreesman suction		1	1	1
- IOS suction tube		1	1	1
- absorbing pads no.4		10	10	10
- compress + sterile gauze		5	5	5
- slings/snuggers		9	9	9
- rough sponge		1	1	1
- tissue glue		2	1	2
- cardioplegia tube		1	1	1
- aortic punch		1	1	1
- bubble tube IOS (5mm)		1	1	1
- steel wire sutures		7	7	7
Surgical disposables				
Surgical drapes	Molnlycke materials, weigh 4.8kg; all consist of (varying) mixtures of low-density polyethylene, viscose, and polypropylene.			
- thorax sheet 200/300cm		1	1	1
- vascular split sheet 200x260		1	1	1
- feet covers 36x28cm		2	2	2
- reinforced table cover		4	4	4
- mayo stand cover		2	2	2
- reinforced mayo stand		1	1	1
- fluid collection pouch		1	1	1
- OP-tape 9x49cm		1	1	1
- adhesive cover sheet 75cm		1	1	1
- camera drape (18x250cm)		1	1	1
Surgical gowns	Molnlycke materials, weigh 0.9–1.0kg; consist of polypropylene material and polyester sleeves.	7	7	8
Cotton gauzes	Combination of X-ray (barium thread) cotton gauzes in 10-packs (10x20cm), one set of gauze swabs (30x30cm), one 5-pack (20x25cm), and one 7.5x500cm ribbon gauze; weigh 0.7–0.8kg	66	66	76
"CABG tray"	Custom, ready-packaged tray of disposables containing: surgical blades (4), 250ml bowl, gauze counting cards (3), crepe paper, diathermic blades (2), instrument cleaner, 24cm kochers (2), lamp cap, needle, needle box 40CT, paper bag, suction tube (5x8mm, 3m), suture booties (2), syringes (3), thorax drain (28-30Ch), cellulose OP towels (4), plastic tray 25x14x5cm, 20cm tube sets (2), mini yankauer (1)	1	1	1
"Wound drain tray"	Custom, ready-packaged tray of disposables containing: cutisoft 10x20cm, end-perforated wound drain, handle XL 9cm, oriflex redon wound drainage system 10Ch, silicone wound rain Ch30, smoke pencil safe air, syringe 50mL	1	1	1
General surgical disposables				
- face masks		8	8	8
- nitrile gloves		62	46	68
- sterile gloves		6	6	6
- theatre caps		8	8	9

- disinfectant<br>- vacuum bag, tube, yankauer		330mL<br>1	330mL<br>1	330mL<br>1
Pleural drain	Pleur-evac (Sahara); largely polycarbonate casing, contains other elements assumed polypropylene	1	1	1
"Endoscopic vessel tray"	Custom, ready-packaged tray of disposables containing: crepe bandages (2), surgical blade, crepe paper, insufflation tube with filter, paper bag	1	1	1
Endoscopic vessel harvesting device	Vasoview 6 Pro	1	1	1
"Suture tray"	Custom, ready-packaged tray of disposables containing: round and pointed beaver, beaver blade, blue clips, needle, titanium clips (2), surgical clamps, surgical sutures (ethibond, mersilene, monocryl, prolene, ticron, vicryl)	1	1	1
Anaesthesia disposables				
Heated air mattress	WarmCloud (long); combination of EVA and (assumed) polypropylene; weighs 0.8 kg	1	1	1
Central venous catheter	Ready-packed insertion set containing: polypropylene sheet, 7Fr double lumen catheter, 9Fr introducer, needles, safety scalpel, skin cover, suture, non-woven cotton gauze,	1	1	1
Urine catheter	Ready-packaged insertion set containing: 14/16Ch catheter, disposable aluminium tray, gauzes for cleaning (3), and (assumed) polycarbonate/HDPE urine collection container (Unometer)	1	1	1
Anaesthesia 'others'<br>- breathing circuit<br>- pressure infusion bag<br>- intubation tube<br>- mayo tube<br>- arterial catheter<br>- i.v. Tegaderm<br>- ECG stickers<br>- art. pressure monitoring syst.<br>- peripheral venous i.v.<br>- central/peripheral therm<br>- BIS Quatro brain monitoring<br>- 11mL instillagel syringe<br>- 3mL syringe<br>- 10mL syringe (flush)<br>- 10mL syringe<br>- 20mL syringe<br>- 50mL syringe<br>- drip Swan I-88<br>- i.v. line E87<br>- i.v. line steritex<br>- blunt filling needle	 *NB: reused over the course of one week*<br>*NB: assumed usage for 50 procedures*	 1/10<br>1/50<br>1<br>1<br>1<br>1<br>10<br>1<br>1<br>1<br>1<br>1<br>2<br>1<br>9<br>2<br>3<br>2<br>2<br>2<br>10	 1/10<br>1/50<br>1<br>1<br>1<br>1<br>10<br>1<br>1<br>1<br>1<br>1<br>2<br>1<br>9<br>2<br>3<br>2<br>2<br>2<br>10	 1/10<br>1/50<br>1<br>1<br>1<br>1<br>10<br>1<br>1<br>1<br>1<br>1<br>2<br>1<br>9<br>2<br>3<br>2<br>2<br>2<br>10

Table D4. Disposable products used per admission (in the ICU)

Product	Additional details	Median (N)	Q1 (N)	Q3 (N)
ICU disposables				
Syringes<br>- 3mL syringe<br>- 10mL syringe<br>- 10mL syringe (flush)<br>- 20mL syringe<br>- 50mL syringe		 3<br>4<br>6<br>1<br>6	 2<br>3<br>3<br>1<br>4	 6<br>6<br>7<br>2<br>9
Nitrile gloves		102	82	144
Volumetric exerciser (Voldyne)		1	1	1
Protective aprons (0.025mm)	Thin polyethylene aprons, worn when seeing a patient	19	13	26
Ventilator-related disposables<br>- suction with Vakon connector<br>- disposable breathing circuit<br>- breathing mask<br>- breathing balloon<br>- breathing circuit HEPA filter		 3<br>1<br>0<br>0<br>1	 2<br>1<br>0<br>0<br>1	 4<br>1<br>1<br>1<br>1
ICU disposables 'others'<br>- Bair Hugger blanket<br>- gauze (10x10cm)<br>- face masks<br>- vacuum bag / tube / yankauer<br>- bed liners 60x60cm<br>- blunt filling needle<br>- luer adapter	 <br>*made of viscose/polyester*	 1<br>30<br>1<br>1<br>1<br>5<br>5	 0<br>16<br>0<br>1<br>0<br>5<br>4	 1<br>45<br>1<br>1<br>2<br>7<br>6

- glucose POC strip		3	3	6
- stitch cutter		2	2	3
- tourniquet		1	1	1
- i.v. line E87		3	3	3
- i.v. 40cm system		2	1	3
- i.v. 150cm system		0	0	1
- drip Swan I-88		2	2	2
- shielded cannula		0	0	2
- combi lock		4	3	7
- disposable washcloth		8	8	8
- oral care kit		0	0	1
- wet wipes		8	7	17
- swab/sampler		0	0	1
- i.v. Tegaderm		2	1	3
- sterile gauze (10x10cm)		5	4	10
- eco care gel (50mL)		1	1	1
- bacillol cleaning wipe		5	5	6
- water bottle (500mL)		2	1	2
- thermometer probe cover		11	9	14
- ECG stickers		9	7	11
- coffee cup + spoon		2	2	2
- vomitus bag		0	0	1
- hair net		1	1	1
- cardboard kidney tray		4	1	5
- tie wraps		8	8	14
- plastic medicine cup		1	1	2

Table D5. Staff and staff commute per CABG trajectory

**Operating Room, staff ***				**Intensive Care Unit, staff ***			
	Median	Q1	Q3		Median	Q1	Q3
Surgeon, resident surgeon	2	2	2	Nursing staff (FTE)	2.1	2.1	2.1
Anaesthetist	1	1	1	Support staff (FTE)	0.6	0.6	0.6
OR nurses	3	3	4	Physicians (FTE)	0.4	0.4	0.4
Nurse anaesthetist(s)	1	1	1	**Intensive Care Unit, staff commute *****			
Perfusionist	1	1	1		Nurse	Support	Physician
**Operating Room, staff commute ****				Travels by car (%)	57	45	10
	Median	Q1	Q3	Car km (per person)	26.5	18.5	26.5
Car km (total)	154	112	189	Travels by bicycle (%)	40	55	90
Bicycle km (total)	20	14	29	Bicycle km (per person)	4.6	5.5	4.6
e-bicycle km (total)	12	6	21	Travels by public transport (%)	3	0	0
				Public transport km (per person)	88	0	0

* For the OR, staff per procedure (surgeons, anaesthetist, OR nurses, nurse anaesthetists, and perfusionists) were counted in the OR – not differentiating in function for transport type; for the ICU, staff per procedure were approximated based on full time equivalents (FTE) of staff involved with the care for one patient per ICU unit – since staff care for multiple patients, varying per time of day (i.e. fewer staff member per patient at night).

** Total staff commute distances for OR staff are one-way commute distances, since only 50% of their commute was allocated to a single procedure (the other half is allocated to the second procedure of the day)

*** Average staff commute distances and percentages were determined via a departmental survey (n=121)

Table D6. Other resources used per CABG trajectory

**Product**	**Additional details**	**Median (N)**	**Q1 (N)**	**Q3 (N)**
Patient travel				
Average car km		97.4	83.2	154.2
Fluid management				
Operating Room<br>- custodiol (mL)<br>- Ringers (1,000 mL)<br>- Fluid bags (NaCl 0.9%, 1&3L)<br>- NaHCO2 (mL, 8.4%)<br>- Gelofusine (mL, 4%)		20<br>2<br>2<br>50<br>3,050<br>350	20<br>1<br>2<br>50<br>3,050<br>350	20<br>2<br>2<br>80<br>3,050<br>350

- Mannitol (mL, 15%)		0	0	1
- packed cells				
Intensive Care Unit				
- NaCl 0.9% (500mL)		2	2	3
- Ringers (250mL)		2	0	4
- Na/glucose 0.45/2.5% (1L)		2	2	2
Reusables				
Operating Room		1	1	1
- Basic CTC set		1	1	1
- ECC set		1	1	1
- Sternotomy set		1	1	1
- Micro set		1	1	1
- IMA spreader		1	1	1
- Magnetic mat				
- Laryngoscope		1	1	1
- Sternal saw		1	1	1
Intensive Care Unit				
- ECG cables		2	2	2
- Blood pressure cuff		1	1	1
- Pulseoximeter cable		1	1	1
- Pressure infusion bag		2	2	2
- Scissors/clamps		2	2	2
- Endocuf test		1	1	1
- Thermometer (ear)		1	1	1
Medication				
Operating Room				
- calciumgluconate (mL, 10%)		10	10	10
- cefazolin (mg)		4,000	2,000	4,000
- dexamethasone (mg)		40	40	40
- heparin (IE/IU)		65,500	57,125	74,875
- midazolam (mg)		323	238	481
- noradrenaline (µg)		27	14	68
- papaverine (IE/IU)		46,500	46,500	46,500
- propofol (mg)		95	15	148
- rocuronium bromide (mg)		100	100	123
- sevoflurane (mL)		4.5	0.5	8.3
- sufentanil (µg)		397	328	455
- tranexaminic acid (mg)		2,898	2,589	3,033
- albumin 20% (100mL)		1	1	1
- protamine (IE/IU)		39,750	34,062	47,311
Intensive Care Unit				
- noradrenaline (mL, 0.1mg/mL)		50	50	50
- Propofol (mL)		0	0	12.5
- morphine (mg)		8.75	4.38	10.00
- ondansetron (mg)		0	0	4
- paracetamol, liquid (mg)		1,500	1,000	3,250
- paracetamol, tablet (mg)		2,000	1,750	2,000
- other drugs (mg) (incl. metoprolol, ASA, pantoprazol)		185	160	244
Long standing medical equipment				
Operating Room				
- IntelliVue Mx800		1	1	1
- Ultrasound		1	1	1
- Anaesthesiology carestation		1	1	1
- Air mattress control unit		1	1	1
- Diathermic control unit		1	1	1
- Luxtec control unit		1	1	1
- ECC-machine		1	1	1
- IOS-machine		1	1	1
- Platform shaker		1	1	1
- Coagulation timer		1	1	1
- Syringe pumps (2 docks)		1	1	1
- Scopic tower		1	1	1
- CO2-insufflator		1	1	1
Intensive Care Unit				
- IntelliVue Mx800		1	1	1
- ventilator		1	1	1
- Syringe pumps (2 docks)		1	1	1
- Bair Hugger unit		1	1	1
- monitoring data terminals (2)		1	1	1
- label scanner		1	1	1
- thermometer		1	1	1
- mobile diagnostic X-ray		1	1	1
Linen				

Operating Room				
- staff OR scrubs		8	8	9
- bed sheet		1	1	1
- blanket		1	1	1
- patient gown		1	1	1
- pillowcase		1	1	1
- thick blanket		1	1	1
- towel		1	1	1
Intensive Care Unit				
- white top/coat + pants		6	6	6
- bed sheet		2	1	4
- blanket		1	1	2
- patient gown		2	1	2
- pillowcase		2	1	3
- towel		4	3	4
Medical gases				
Operating Room				
- O2		273	203	313
- CO2		46.3	31.5	76.2
- Compressed air		335	305	345
Intensive Care Unit				
- O2 (ventilator)		388	298	613
- O2 (nasal)		1,620	1,114	2,892
Others				
Operating Room				
- hemocytometry		3	3	3
- coagulation tests		6	6	6
- blood proteins / renal		1	1	1
- arterial blood gas		4	4	4
Intensive Care Unit				
- hemocytometry		2	2	3
- coagulation tests		2	2	2
- blood proteins / renal		3	3	4
- arterial blood gas		2	2	3
- POC glucose		3	3	6

Table D7. OR energy consumption, reference table

Category	Energy requirement (per hour)		Energy source
**HVAC in the OR**			
Ventilation fresh outside air	0.5 kWh		
Ventilation air recirculation	1.4 kWh		
Ventilation air outgoing	0.3 kWh		
Heating of fresh outside air *	3.0 kWh		Electricity use
Cooling/dehumidification of fresh outside air *	1.0 kWh		
Heating of air (after dehumidifcation) *	0.4 kWh		
Cooling of air (recirculation)	6.5 kWh		
**Total for HVAC**	**13.1 kWh**		
Humidification of fresh outside air (using steam)	10.2 MJ		Natural gas
**Category**	**Energy requirement (per hour)**		**Information source**
**Lighting in the OR**			
Ceiling lighting (3x18W luminescent tube fixtures)	54 Wh		in-house energy expert
	Active	Standby	
**Equipment in the OR ****			
Screens and PCs	0.3 kWh	0.2 kWh	
- Screens + monitoring screens (7)	*18.8 Wh*	-	'Werkblad Groene IC'
- IntelliVue Mx800 monitor (1)	*35 Wh*	-	'Werkblad Groene IC'
- Computer on wheels (1)	*30 Wh*	*17 Wh*	'Werkblad Groene IC'
- Widescreen (TV) on OR wall (1)	*100 Wh*	-	energysage.com
General OR	0.5 kWh	0.5 kWh	
- warming cabinet (medicalexpo GE-2350S) (1)	*150 Wh*	-	product manual
- refrigerator (1)	*250 Wh*	-	assumed
- operating lights marLED (2)	*40 Wh*	-	product manual
- OR table TruSystem (1)	-	-	*assumed negligible*

Anaesthesia	0.6 kWh	0.2 kWh	
- Carestation GE Avance CS2 (1) (used proxy)	*70 Wh*	*65 Wh*	Drinhaus et al, 2023
- Syringe pump (8)	*22W*	-	'Werkblad Groene IC'
- Ultrasound GE Vivid E90 (1)	*500W*	-	davismedical manual
- Defib Efficia DFM100 (1)	-	-	*not in use*
Heated air mattress (KanMed warmcloud) (1)	0.4 kWh	-	product manual
Perfusion	1.2 kWh	0.2 kWh	
- HLM Stockert/Sorin S5 (1)	*800 Wh*	-	S5 LivaNova manual
- heater/cooler unit (1)	-	-	*not in use*
- CellSaver Sorin Xtra (average, used proxy) (1)	*220 Wh*	-	Autolog IQ manual
- platform shaker Titramax2000 (1)	*31 Wh*	-	profilab24 technical details
- GEM Premier5000 blood gas system (1)	*150 Wh*	-	sfgh-poct manual
- ACT Plus coagulation timer (1)	*40 Wh*	-	indiamart.com
NB: assumed 10min/h use for blood test systems			
Surgical	1.3 kWh	0.2 kWh	
- headlamp Luxtec MLX (incl. power dissipation) (2)	*450 Wh*	-	product manual
- scopic tower: Image S X-link TC301 (1)	*62 Wh*	-	Karl Storz website
- scopic tower: endoflator40 UI400 (40L/min) (1)	*90 Wh*	-	Karl Storz website
- scopic tower: powerLED Rubina TL400 (1)	*220 Wh*	-	Karl Storz website
- diathermy set ERBE ICC50 (1)	-	-	*assumed negligible*
Electricity use unaccounted for	0.1 kWh	0.1 kWh	own assumption

** Representing an OR's annual average, both heating and cooling of fresh outside air were included, whereas in reality either of the two would be taking place (depending on outside air conditions).*
*** Equipment energy requirements per hour were approximated per user group (non-italic, indicated in kWh), based on the sum of the individual devices in use (italic, indicated in Wh). Since device groups were mostly unplugged when no surgery was performed (e.g. ultrasounce, scopic tower), we approximated the groups' accumulated 'Standby' energy use based on usage patterns.*

Table D8. OR energy consumption, calculation table (for mean values)

Category	Calculation	Total
**HVAC**		
Ventilation/heating/cooling	13.1 kWh*ORD + 13.1 kWh*periOR	86.7 kWh
Humidification	10.2 MJ*ORD + 10.2 MJ*periOR	67.5 MJ
**Lights**		
Ceiling lights (10)	10*Active*ORD + 7*Active*periOR	3.3 kWh
**Equipment in the OR**		
Screens and PCs	Active*ORD + Standby*periOR	1.8 kWh
General OR	Active*ORD + Active*perioOR	3.3 kWh
Anaesthesia	Active*ORD + Standby*periOR	3.2 kWh
Heated air mattress	Active*ORD	2.0 kWh
Perfusion	Active*ORD + Standby*periOR	5.9 kWh
Surgical	Active*ORD * Standby*periOR	6.4 kWh
Unaccounted	Active*ORD + Active*periOR	0.7 kWh
**Total**		**23.3 kWh**
Parameters: ORD (OR duration) = 4.62h; periOR (allocated perioperative time) = 2h		

Table D9. ICU energy consumption, reference table

Category	Energy requirement (per hour)	Energy source
**HVAC in the ICU**		
Ventilation fresh outside air	0.5 kWh	
Ventilation air outgoing	0.14 kWh	
Heating of fresh outside air *	1.69 kWh	Electricity use
Cooling/dehumidifcation of fresh outside air *	0.14 kWh	

Category	Energy requirement (per hour)		Information source
**Total for HVAC**	**2.47 kWh**		
Humidifcation of fresh outside air (using steam)	4.67 MJ		Natural gas
**Category**	**Energy requirement (per hour)**		**Information source**
	Active	Standby	
**Equipment inside patient room**			
Ventilator (Maquet, Servo-i)	38 Wh	33 Wh	product manual
Infusor pump	4.6 Wh	3.4 Wh	Drinhaus et al, 2023
Syringe pump, in use	4.4 Wh	2.4 Wh	Drinhaus et al, 2023
Vitals monitor	30 Wh	-	'Werkblad Groene IC'
Vitals data transfer terminal (Datalogic)	7 Wh	-	product manual
Bedside computer	30 Wh	17 Wh	'Werkblad Groene IC'
Patient bed	22 Wh	0.5 Wh	'Werkblad Groene IC'
Forced heating blanket (Bair Hugger)	800 Wh	-	product manual
Electricity use unaccounted for	30 Wh	-	own assumption
	Active	Standby	
**Equipment + lights outside patient room**			
Computer, shared workstation	30 Wh	17 Wh	'Werkblad Groene IC'
Vitals monitoring screen	18.8 Wh	-	Drinhaus et al, 2023
Vitals data transfer terminal (Datalogic)	7 Wh	-	product manual
Lights (2x18W luminescent tube fixtures)	36 Wh	-	in-house energy expert
Urinal cleaning machine	250Wh	-	'Werkblad Groene IC'
Electricity use unaccounted for	90 Wh	-	own assumption

** Representing an ICU's annual average, both heating and cooling of fresh outside air were included, whereas in reality either of the two would be taking place (depending on outside air conditions).*

Table D10. ICU energy consumption, calculation table (for mean values)

Category	Calculation	Total
**HVAC**		
Ventilation/heating/cooling	2.47 kWh*LOS	54.5 kWh
Humidifcation	4.67 MJ*LOS	103.0 MJ
**Inside patient room**		
Ventilator (1)	Active*ventilator + Standby*(LOS-ventilator)	772 Wh
Infusor pump (1)	Active*infusor + Standby*(LOS-infusor)	88.3 Wh
Syringe pump, in use (2)	2*Active*pump + 2*Standy*(LOS-pump)	122 Wh
Pump, not in use (2)	2*Standby*LOS	128 Wh
Vitals monitor (1)	Active*LOS	662 Wh
Vitals terminal (2)	2*Active*LOS	309 Wh
Bedside computer (2)	2*Active*1h + 2*Standby*(LOS-1h)	777 Wh
Patient bed (1)	Active*1h + Standby*(LOS-1h)	32.5 Wh
Forced heating blanket (1)	1 (=1 hour of active time)	800 Wh
Unaccounted (1)	1*LOS	662 Wh
**Total**		**4.4 kWh**
**Outside patient room**		
Computers (3)	3*allocation*Active*3h + 3*allocation*Standby*(LOS–3h)	207 Wh
Vitals monitor (2)	2*allocation*Active*LOS	138 Wh
Vitals terminal (2)	2*allocation*Active*LOS	51.5 Wh
Lights, nursing station (2)	2*allocation*Active*LOS	265 Wh
Lights, hallway (4)	4*allocation*(LOS*day_only)	353 Wh
Urinal cleaning (1)	1 (=single cycle)	250 Wh
Unaccounted (1)	1*allocation*LOS	331 Wh
**Total**		**1.6 kWh**
Parameters: LOS = 22.05h; ventilator = 8.77h (mean time that patient is on the ventilator); pump = 4h (mean time that patients received propofol or norepinephrine in the ICU); infusor = 50% of LOS (assumed based on observations); allocation = 1/6th (= average bed occupation of respective ICU unit); day_only = 2/3 (=switched off at night, 1/3rd of the time)		

Supplement S5 - Detailed environmental impact assessment results

Table E1. Endpoint results (H)

Characterization method: ReCiPe 2016 Endpoint (H) V1.08 / World (2010) H/A [recalculated dd16072024]				
**CABG + ICU median**	**DALYs**	**(%)**	**Ecosystems**	**(%)**
Total	8,10E-04	100	1,86E-06	100
Global warming, human health	3,84E-04	47	-	-
Global warming, terrestrial ecosystems	-	-	1,16E-06	62
Global warming, freshwater ecosystems	-	-	3,17E-11	0
Stratospheric ozone depletion	2,14E-07	0	-	-
Ionizing radiation	1,21E-07	0	-	-
Ozone formation, human health	7,24E-07	0	-	-
Fine particulate matter formation	2,43E-04	30	-	-
Ozone formation, terrestrial ecosystems	-	-	1,09E-07	6
Terrestrial acidification	-	-	1,98E-07	11
Freshwater eutrophication	-	-	7,50E-08	4
Marine eutrophication	-	-	1,09E-10	0
Terrestrial ecotoxicity	-	-	1,85E-08	1
Freshwater ecotoxicity	-	-	1,74E-08	1
Marine ecotoxicity	-	-	3,45E-09	0
Human carcinogenic toxicity	8,47E-05	10	-	-
Human non-carcinogenic toxicity	8,94E-05	11	-	-
Land use	-	-	2,08E-07	11
Water consumption, human health	8,26E-06	1	-	-
Water consumption, terrestrial ecosystem	-	-	7,28E-08	4
Water consumption, aquatic ecosystem	-	-	1,10E-11	0
**CABG + ICU Q25**	**DALYs**		**Ecosystems**	
Total	7,60E-04		1,73E-06	
**CABG + ICU Q75**	**DALYs**		**Ecosystems**	
Total	9,04E-04		2,06E-06	

Table E2. Midpoint results (H) for selected impacts (meeting the ≥10% damage cut-off for human health or ecosystems in Table D1)

	**Non-carcinogenic toxicity** (kg 1,4-dichlorobenzene equitvalents)	**Carcinogenic toxicity** (kg 1,4-dichlorobenzene equivalents)	**Land use** (m2 annual crop equivalents)	**Terrestrial acidification** (kg SO2 equivalents)	**Fine particulate matter formation** (kg PM2.5 equivalents)	**Global warming** (kg CO2 equivalents)
OR + ICU (**Q25**)	370	23,7	22	0,878	0,363	383
2.5% (Q25)	-7,23E+04	-86,6	16,3	0,809	0,331	359
97.5% (Q25)	7,54E+04	133	28,2	0,971	0,404	410
OR + ICU (**median**)	392	25,5	23,5	0,936	0,387	414
OR + ICU (**Q75**)	434	28,6	26,3	1,04	0,429	461
2.5% (Q75)	-8,24E+04	-90,5	19,6	0,945	0,388	431
97.5% (Q75)	8,10E+04	149	33,8	1,14	0,475	498
OR (**median**)	311	19,4	18,5	0,733	0,302	314
ICU (**median**)	56,4	3,82	3,23	0,147	0,0607	77,3
Patient travel (**median**)	24,6	2,32	1,78	0,0549	0,0236	22,6
OR (**Q25**)	298	18,3	17,6	0,702	0,289	296
OR (**Q75**)	324	20,5	19,7	0,768	0,316	333
ICU (**Q25**)	51	3,45	2,86	0,129	0,0531	67,9
ICU (**Q75**)	71,3	4,49	3,85	0,182	0,0752	92,6
Patient transport (**Q25**)	21	1,98	1,52	0,0469	0,0201	19,3
Patient transport (**Q75**)	38,9	3,67	2,82	0,0869	0,0373	35,8

Supplement S6 – Contribution analysis of environmental impacts, sensitivity analysis, and mitigation scenarios

Table F1. Contribution analysis for all 18 midpoint impacts

Impact category	Unit	Total	Total %	Disposables OR	Disposables OR	Energy use ICU+ OR	Energy use	Employee transportation	Employee commute	Disposables ICU	Disposables ICU	Patient transportation	Patient travel	Fluid management	Fluid management	Medication	Medication	Devices OR + ICU	Devices OR + ICU	Reusables	Reusables	Linen	Linen	Medical gasses	Medical gasses	Others	Others
Global warming	kg CO2 eq	413,81769	100	162,2148	39,2%	78,864164	19,1%	54,63516	13,2%	13,193223	3,2%	22,624447	5,5%	20,782676	5,0%	18,088691	4,4%	8,1285959	2,0%	21,345325	5,2%	6,2206586	1,5%	3,5855156	0,9%	4,1344369	1,0%
Stratospheric ozone depletion	kg CFC11 eq	4,03E-04	100	0,0001807	44,8%	2,4255E-05	6,0%	4,0669E-05	10,1%	8,8938E-06	2,2%	1,71E-05	4,2%	0,00010675	26,5%	0	0,0%	3,63E-06	0,9%	5,91E-06	1,5%	9,96E-06	2,5%	1,56E-06	0,4%	3,65E-06	0,9%
Ionizing radiation	kBq Co-60 eq	14,213807	100	8,1981624	57,7%	0,31345033	2,2%	1,43953781	10,1%	0,71578186	5,0%	0,60166747	4,2%	0,54718326	3,8%	0	0,0%	0,72011841	5,1%	0,33119889	2,3%	0,10154379	0,7%	1,1587052	8,2%	0,08645754	0,6%
Ozone formation, Human health	kg NOx eq	0,79502872	100	0,34015544	42,8%	0,11543853	14,5%	0,12817701	16,1%	0,03452814	4,3%	0,05292846	6,7%	0,04234165	5,3%	0	0,0%	0,02607903	3,3%	0,02070964	2,6%	0,008605578	1,1%	0,00588238	0,7%	0,02018287	2,5%
Fine particulate matter	kg PM2.5 eq	0,38671543	100	0,19772711	51,1%	0,03348813	8,7%	0,05732866	14,8%	0,0153234	4,0%	0,02355146	6,1%	0,01791653	4,6%	0	0,0%	0,01891035	4,9%	0,00958568	2,5%	0,004341274	1,1%	0,00380622	1,0%	0,00473662	1,2%
Ozone formation, Terrestrial ecosystems	kg NOx eq	0,84704925	100	0,35984629	42,5%	0,12069806	14,2%	0,14141444	16,7%	0,03641512	4,3%	0,05846666	6,9%	0,04493355	5,3%	0	0,0%	0,02682267	3,2%	0,02214134	2,6%	0,009174338	1,1%	0,00627219	0,7%	0,02086459	2,5%
Terrestrial acidification	kg SO2 eq	0,93562677	100	0,48081517	51,4%	0,09301936	9,9%	0,13354858	14,3%	0,03479993	3,7%	0,05493546	5,9%	0,04556354	4,9%	0	0,0%	0,03931799	4,2%	0,02087366	2,2%	0,011993256	1,3%	0,00913433	1,0%	0,01162549	1,2%
Freshwater eutrophication	kg P eq	0,11194665	100	0,05187619	46,3%	0,00185979	1,7%	0,0111809	10,0%	0,00340416	3,0%	0,00459073	4,1%	0,00366284	3,3%	0	0,0%	0,01033393	9,2%	0,01113832	9,9%	0,011554876	10,3%	0,00189541	1,7%	0,00044495	0,4%
Marine eutrophication	kg N eq	6,44E-02	100	0,04782286	74,3%	0,00023742	0,4%	0,00593147	9,2%	0,00045277	0,7%	0,0024916	3,9%	0,00080691	1,3%	0	0,0%	0,0004761	0,7%	0,00124	1,9%	0,004612557	7,2%	0,00018373	0,3%	0,00013	0,2%

																				955						288	
Terrestrial ecotoxicity	kg 1,4-DCB	1619,5938	100	738,25937	45,6%	83,964962	5,2%	343,84332	21,2%	42,821686	2,6%	143,18616	8,8%	71,911629	4,4%	0	0,0%	91,237468	5,6%	41,994459	2,6%	13,997096	0,9%	18,363673	1,1%	30,013994	1,9%
Freshwater ecotoxicity	kg 1,4-DCB	25,087377	100	10,937035	43,6%	0,0332175	0,1%	5,3909714	21,5%	0,71583866	2,9%	2,2220859	8,9%	0,37222401	1,5%	0	0,0%	4,734274	18,9%	0,31013491	1,2%	0,11932575	0,5%	0,1539552	0,6%	0,09831511	0,4%
Marine ecotoxicity	kg 1,4-DCB	32,775864	100	14,256273	43,5%	0,10145036	0,3%	6,9024736	21,1%	0,95710364	2,9%	2,8421618	8,7%	0,52258575	1,6%	0	0,0%	6,2984571	19,2%	0,41988353	1,3%	0,12938139	0,4%	0,20587504	0,6%	0,14021904	0,4%
Human carcinogenic toxicity	kg 1,4-DCB	25,522822	100	11,505288	45,1%	0,48090015	1,9%	5,6964477	22,3%	0,73085541	2,9%	2,3183848	9,1%	1,3941921	5,5%	0	0,0%	1,2038243	4,7%	1,6125915	6,3%	0,14680498	0,6%	0,21872734	0,9%	0,2148066	0,8%
Human non-carcinogenic toxicity	kg 1,4-DCB	391,95643	100	183,95611	46,9%	10,1958196	2,6%	61,254547	15,6%	13,761902	3,5%	24,568046	6,3%	8,7494294	2,2%	0	0,0%	75,45051	19,2%	5,5972288	1,4%	2,4471149	0,6%	3,2930157	0,8%	2,6827069	0,7%
Land use	m2a crop eq	23,474279	100	12,371228	52,7%	1,08548795	4,6%	4,2476018	18,1%	0,53452982	2,3%	1,7796455	7,6%	0,30507922	1,3%	0	0,0%	0,35317805	1,5%	1,3864163	5,9%	1,178408	5,0%	0,10004145	0,4%	0,13266299	0,6%
Mineral resource scarcity	kg Cu eq	1,7164396	100	0,64830177	37,8%	0,08518211	5,0%	0,37013727	21,6%	0,03031548	1,8%	0,15126504	8,8%	0,02025118	1,2%	0	0,0%	0,30094375	17,5%	0,08409286	4,9%	0,008307286	0,5%	0,0083178	0,5%	0,00932504	0,5%
Fossil resource scarcity	kg oil eq	134,47596	100	56,465086	42,0%	29,074252	21,6%	16,1388148	12,0%	5,2677926	3,9%	6,6856408	5,0%	6,8040451	5,1%	0	0,0%	2,0559048	1,5%	7,6010415	5,7%	1,9978662	1,5%	1,0199043	0,8%	1,3656076	1,0%
Water consumption	m3	8,4400566	100	5,5531294	65,8%	0,3684255	4,4%	0,36522376	4,3%	0,08285496	1,0%	0,1532359	1,8%	0,1626279	1,9%	0	0,0%	0,06851621	0,8%	1,1296722	13,4%	0,42225676	5,0%	0,11481001	1,4%	0,019304	0,2%

Table F2. Contribution analysis for OR and ICU energy use and employee commute (specification of Table F1)

Impact category	Unit	Energy OR	Energy OR	Energy ICU	Energy ICU	Employee transport OR		Employee transport ICU	
Global warming	kg CO2 eq	47,603197	11,5%	31,260967	7,6%	36,276454	8,8%	18,358706	4,4%
Stratospheric ozone depletion	kg CFC11 eq	1,53E-05	3,8%	8,98E-06	2,2%	2,71E-05	6,7%	1,35E-05	3,4%
Ionizing radiation	kBq Co-60 eq	0,19324235	1,4%	0,12020798	0,8%	0,97157832	6,8%	0,46795949	3,3%
Ozone formation, Human health	kg NOx eq	0,07282823	9,2%	0,0426103	5,4%	0,08500173	10,7%	0,04317528	5,4%
Fine particulate matter	kg PM2.5 eq	0,02117251	5,5%	0,01231562	3,2%	0,0382395	9,9%	0,01908916	4,9%
Ozone formation, Terrestrial ecosystems	kg NOx eq	0,07604321	9,0%	0,04465485	5,3%	0,0938109	11,1%	0,04760354	5,6%
Terrestrial acidification	kg SO2 eq	0,05889822	6,3%	0,03412114	3,6%	0,08901654	9,5%	0,04453204	4,8%
Freshwater eutrophication	kg P eq	0,00115432	1,0%	0,00070547	0,6%	0,00748762	6,7%	0,00369329	3,3%
Marine eutrophication	kg N eq	0,00014858	0,2%	8,88E-05	0,1%	0,00396838	6,2%	0,00196309	3,0%
Terrestrial ecotoxicity	kg 1,4-DCB	53,30197	3,3%	30,662992	1,9%	229,96988	14,2%	113,87344	7,0%
Freshwater ecotoxicity	kg 1,4-DCB	0,01678205	0,1%	0,01643545	0,1%	3,613777	14,4%	1,7771944	7,1%
Marine ecotoxicity	kg 1,4-DCB	0,05819865	0,2%	0,04325172	0,1%	4,6273388	14,1%	2,2751348	6,9%
Human carcinogenic toxicity	kg 1,4-DCB	0,27136685	1,1%	0,2095333	0,8%	3,8048763	14,9%	1,8915714	7,4%
Human non-carcinogenic toxicity	kg 1,4-DCB	6,4316911	1,6%	3,7641285	1,0%	41,069096	10,5%	20,185451	5,1%
Land use	m2a crop eq	0,68650748	2,9%	0,39898047	1,7%	2,8322191	12,1%	1,4153827	6,0%
Mineral resource scarcity	kg Cu eq	0,05301252	3,1%	0,03216959	1,9%	0,24749516	14,4%	0,12264211	7,1%
Fossil resource scarcity	kg oil eq	17,438728	13,0%	11,635524	8,7%	10,704586	8,0%	5,4342288	4,0%
Water consumption	m3	0,18041229	2,1%	0,18801321	2,2%	0,24570843	2,9%	0,11951533	1,4%

Table F3. Contribution analysis for OR disposables

Impact category	Unit	Total	Top	**Total disposables**	ECC set	ECC set %	Cells aver set	Cellsaver set %	Perfusionist disposables	Perfusionist disposables %	CABG tray	CABG tray %	Gang A tray	Gang A tray %	Anesthesia disposables	Anesthesia disposables %	Endoscopic vein tray	Endoscopic vein tray %	General surgical disposables	General surgical disposables %	Disposable kidney tray	Disposable kidney tray %	Pleural drain	Pleural drain %	EVH device	EVH device %
Global warming	kg CO_2 eq	313,87476	115,38351	**162,21**	55,283456	34,1	11,123529	6,9	4,0056334	2,5	40,538834	25,0	11,659842	7,2	14,719103	9,1	5,5379411	3,4	13,691131	8,4	1,3115568	0,8	2,3682615	1,5	1,9755118	1,2
Stratospheric ozone depletion	kg CFC11 eq	0,00032104	0,00011321	**0,00**	4,44E-05	24,5	6,03E-06	3,3	3,58E-06	2,0	9,07E-05	50,2	1,41E-05	7,8	7,23E-06	4,0	5,07E-06	2,8	4,35E-06	2,4	2,66E-06	1,5	1,81E-06	1,0	9,08E-07	0,5
Ionizing radiation	kBq Co-60 eq	11,104512	1,9347712	**8,20**	3,4971598	42,7	0,77591348	9,5	0,15235107	1,9	1,6687908	20,4	0,50657671	6,2	0,60600394	7,4	0,16677427	2,0	0,58976513	7,2	5,15E-02	0,6	0,10461728	1,3	0,07869251	1,0
Ozone formation, Human health	kg NOx eq	0,60067432	0,17551715	**0,34**	0,0915926	26,9	0,02087426	6,1	0,00759298	2,2	0,10684735	31,4	0,02583562	7,6	0,02877377	8,5	0,01030232	3,0	0,0321235	9,4	0,00309634	0,9	0,00619597	1,8	0,00692073	2,0
Fine particulate matter formation	kg PM2.5 eq	0,30586935	0,06990274	**0,20**	0,05409676	27,4	0,01295626	6,6	0,00416257	2,1	0,06232947	31,5	0,02092573	10,6	0,01645033	8,3	0,00550664	2,8	0,01346155	6,8	0,00203728	1,0	0,00328117	1,7	0,00251935	1,3
Ozone formation, Terrestrial ecosystems	kg NOx eq	0,63747622	0,18381902	**0,36**	0,0980801	27,3	0,02229462	6,2	0,00793655	2,2	0,11194573	31,1	0,02739513	7,6	0,03033372	8,4	0,01083318	3,0	0,03395855	9,4	0,00326246	0,9	0,00652994	1,8	0,00727632	2,0
Terrestrial acidification	kg SO_2 eq	0,74030715	0,17047544	**0,48**	0,12125605	25,2	0,02735609	5,7	0,01001174	2,1	0,16745379	34,8	0,05257231	10,9	0,03881664	8,1	0,01439233	3,0	0,03259209	6,8	0,00364015	0,8	0,00683939	1,4	0,00588459	1,2
Freshwater eutrophication	kg P eq	0,0914683	0,0321045	**0,05**	0,01494577	28,8	0,0043302	8,3	0,00099074	1,9	0,01505836	29,0	0,00511097	9,9	0,00468564	9,0	0,00144854	2,8	0,00325858	6,3	0,00043198	0,8	0,00084569	1,6	0,00076973	1,5
Marine eutrophication	kg N eq	0,05618435	0,00439311	**0,05**	0,0016286	3,4	0,0005178	1,1	0,00141185	3,0	0,03924076	82,1	0,00037645	0,8	0,00051609	1,1	0,00350857	7,3	0,000397	0,8	4,79E-05	0,1	5,97E-05	0,1	0,00011808	0,2

Terrestrial ecotoxicity	kg 1,4-DCB	1245,9307	277,70146	**738,26**	185,69362	25,2	52,162826	7,1	10,809194	1,5	130,00989	17,6	186,2319	25,2	91,880415	12,4	15,934886	2,2	40,896723	5,5	9,9438761	1,3	6,9767887	0,9	7,7192487	1,0
Freshwater ecotoxicity	kg 1,4-DCB	20,043904	5,4930924	**10,94**	2,4603864	22,5	0,64945787	5,9	0,1746314	1,6	2,5732678	23,5	2,2467496	20,5	1,5743973	14,4	0,28554924	2,6	0,66498803	6,1	0,08307804	0,8	0,08308883	0,8	0,14144018	1,3
Marine ecotoxicity	kg 1,4-DCB	26,261198	7,377586	**14,26**	3,2495099	22,8	0,84707005	5,9	0,22986048	1,6	3,2392751	22,7	2,9192207	20,5	2,0826144	14,6	0,37314811	2,6	0,91356938	6,4	0,1141507	0,8	0,10790638	0,8	0,17994777	1,3
Human carcinogenic toxicity	kg 1,4-DCB	19,711804	4,4016398	**11,51**	4,5680426	39,7	1,4259483	12,4	0,20679448	1,8	2,0002679	17,4	0,83836012	7,3	0,94360425	8,2	0,1722872	1,5	0,4881061	4,2	0,49605917	4,3	0,18863658	1,6	0,17718094	1,5
Human non-carcinogenic toxicity	kg 1,4-DCB	322,96299	97,937784	**183,96**	40,464475	22,0	9,4548669	5,1	3,3825131	1,8	47,185099	25,7	35,504404	19,3	25,734001	14,0	4,2798199	2,3	13,092823	7,1	1,3942127	0,8	1,770705	1,0	1,6931952	0,9
Land use	m2a crop eq	18,533134	3,3296868	**12,37**	1,4035567	11,3	0,4145012	3,4	0,26800975	2,2	7,2082902	58,3	0,72902546	5,9	0,40251695	3,3	0,58720076	4,7	0,88965338	7,2	0,11092156	0,9	0,04859717	0,4	0,30895457	2,5
Mineral resource scarcity	kg Cu eq	1,3633164	0,46751945	**0,65**	0,20779442	32,1	0,06826946	10,5	0,00925325	1,4	0,10449526	16,1	0,1057913	16,3	0,07865155	12,1	0,00811363	1,3	0,02464318	3,8	0,02745902	4,2	0,00577492	0,9	0,00805579	1,2
Fossil resource scarcity	kg oil eq	101,68897	34,519295	**56,47**	20,082608	35,6	4,3038765	7,6	1,197139	2,1	11,862411	21,0	3,77839	6,7	5,5617399	9,8	1,6654894	2,9	5,8642327	10,4	0,41880427	0,7	0,85665574	1,5	0,87373957	1,5
Water consumption	m3	7,4812289	1,6823911	**5,55**	0,49826454	9,0	0,07504407	1,4	0,15234742	2,7	4,1525043	74,8	0,08628216	1,6	0,09906324	1,8	0,37766056	6,8	0,07137719	1,3	0,00783389	0,1	0,02301516	0,4	0,00973693	0,2

Table F4. Contribution analysis of ICU disposables

Impact category	Unit	Total	Top	Nitrile gloves	Forced heating blanket	Syringes	Ventilator disposables	Protective aprons	i.v. lines and disposables	Patient washing	Room cleaning/ desinfecting	Water bottles	Bandage material	Sharps / blood drawing	Volumetric exerciser	Gauzes	Vacuum bag / yankauer	Absorbent underpad	Vitals - thermometer / ECG
Global warming	kg CO2 eq	13,193223	0,84982681	1,6569353	1,0678528	1,821591	1,0932865	1,2294725	0,53188367	1,0200347	0,27277908	0,40960101	0,11551685	0,09150874	1,5375874	0,16621605	1,1047194	0,11058103	0,1138303
Stratospheric ozone depletion	kg CFC11 eq	8,89E-06	3,06E-07	5,78E-07	1,41E-07	1,74E-06	2,34E-07	3,52E-07	6,40E-07	1,55E-06	4,73E-07	1,26E-06	1,76E-07	1,75E-07	3,50E-07	2,73E-07	3,60E-07	6,33E-08	2,17E-07
Ionizing radiation	kBq Co-60 eq	0,71578186	0,02269191	0,21326566	0,02304217	0,06306217	0,03697399	0,05614722	0,03610656	0,06119235	0,00740892	0,0318879	0,00814684	0,00442881	0,07061562	0,0094469	0,05850623	0,00807836	0,00478026
Ozone formation, Human health	kg NOx eq	0,03452814	0,00436532	0,00502858	0,00164765	0,00499222	0,00219654	0,00391251	0,00099658	0,00298586	0,00050572	0,00078625	0,00029003	0,00017443	0,00384382	0,00057769	0,00164202	0,0002912	0,00029171
Fine particulate matter formation	kg PM2.5 eq	0,0153234	0,00102269	0,00225886	0,00083201	0,00227023	0,00100634	0,00152348	0,00055885	0,00152491	0,00025428	0,00037571	0,00021901	0,00012834	0,00176985	0,00035542	0,00084698	0,00023136	0,00014507
Ozone formation, Terrestrial ecosystems	kg NOx eq	0,03641512	4,51E-03	0,0054297	1,75E-03	5,23E-03	0,00231277	4,14E-03	1,06E-03	3,11E-03	5,52E-04	0,00083854	3,10E-04	1,85E-04	4,05E-03	0,00060052	0,00173903	0,00029936	0,00030396
Terrestrial acidification	kg SO2 eq	0,03479993	0,00255314	0,00513858	0,00202488	0,00500862	0,00235515	0,00350735	0,0012999	0,00333128	0,00061384	0,0008732	0,00052451	0,00023166	0,00389511	0,00084266	0,00197206	0,00031254	0,00031545
Freshwater eutrophication	kg P eq	0,00340416	0,00010161	0,00054899	0,00015561	0,00046067	0,00021686	0,00031439	0,00017064	0,00036697	5,87E-05	0,00013291	5,54E-05	2,70E-05	0,00042327	6,86E-05	0,00021522	5,68E-05	3,05E-05
Marine eutrophication	kg N eq	0,00045277	3,11E-05	6,69E-05	1,35E-05	3,82E-05	3,76E-05	3,65E-05	1,58E-05	9,89E-05	5,43E-06	1,74E-05	6,77E-06	2,57E-06	3,48E-05	8,01E-06	2,47E-05	8,77E-06	5,83E-06
Terrestrial ecotoxicity	kg 1,4-DCB	42,821686	6,4954606	5,3026904	2,9499081	3,4258117	2,8286819	2,1873075	1,5824024	4,6675256	0,64708925	2,1824585	0,44548426	0,57593445	4,2327501	0,71209392	3,937098	0,2780478	0,37094122
Freshwater ecotoxicity	kg 1,4-DCB	0,71583866	0,02627469	0,15650512	0,04330615	0,04827779	0,06084853	0,11042554	0,01895726	0,05045262	0,00988351	0,01651548	0,00665467	0,00420169	0,07852003	0,01441019	0,04482579	0,01674864	0,00903096
Marine ecotoxicity	kg 1,4-DCB	0,95710364	0,03678152	0,20641642	0,06009876	0,06526096	0,08286386	0,14445974	0,02545012	0,06635711	0,01314083	0,02288128	0,00882586	0,00574961	0,10414045	0,01902501	0,06186189	0,02191347	0,01187676
Human carcinogenic toxicity	kg 1,4-DCB	0,73085541	0,0435655	0,09533636	0,02921976	0,16516049	0,03845252	0,05241461	0,05524498	0,05910395	0,01044182	0,0178687	0,00602329	0,02742471	0,05844481	0,010203	0,04233409	0,00668129	0,01293554
Human non-carcinogenic toxicity	kg 1,4-DCB	13,761902	0,60735309	3,2484907	0,59324561	1,1450637	0,95218023	2,1029413	0,41308421	0,98165605	0,19861421	0,31262016	0,14577857	0,0835682	1,4633718	0,29382279	0,73074854	0,32287584	0,16648694
Land use	m2a crop eq	0,53452983	0,03926501	0,11181682	0,0085448	0,0534876	0,03718597	0,03115186	0,03301392	0,02110262	0,00313382	0,01078558	0,02905877	0,00309051	0,02825053	0,03256252	0,0265659	0,06330695	0,00220667
Mineral resource scarcity	kg Cu eq	0,03031548	0,00188064	0,00536234	0,00144395	0,00366614	0,00159546	0,00184145	0,00126854	0,0036958	0,00052233	0,00115377	0,00028179	0,00127358	0,00295764	0,00053978	0,00210472	0,00017423	0,00055332
Fossil resource scarcity	kg oil eq	5,2677926	0,27580155	0,73973529	0,47794439	0,79469902	0,39897814	0,60611192	0,22025249	0,43062268	0,0988077	0,15265318	0,04333574	0,03294119	0,56205117	0,04541547	0,331244	0,02121277	0,03598588

Water consumption	m3	0,08285496	0,00424507	0,02003399	0,00235577	0,00996274	0,00593129	0,00853197	0,00177383	-0,00363675	0,00174949	0,00436454	0,00144771	0,00059194	0,01114212	0,00187426	0,0110658	0,00072245	0,00069875

Table F5. Contribution analysis of individual disposables (underlying manuscript Figure 6)

Category	kg CO2 eq	% kg CO2 eq	m2a land eq	% m2a land eq	kg PM2.5 eq	%kg PM2.5 eq	kg SO2 eq	%kg SO2 eq
Extracorporeal circulation set	55,3	31,5	1,4035567	10,9	0,05409676	25,4	0,12125605	23,5
Patient, device and, table covers	36	20,5	2,27	17,6	0,0477	22,4	0,111	21,5
Intraoperative salvage set	11,1	6,3	0,4145012	3,2	0,01295626	6,1	0,02735609	5,3
Surgical gowns	10,0	5,7	0,697	5,4	0,0095	4,5	0,0232	4,5
Cotton gauzes	9,5	5,4	5,34	41,4	0,0199	9,3	0,0686	13,3
Disposable heated air mattress	6,0	3,4	0,0819	0,6	0,00438	2,1	0,0103	2,0
Endoscopic vessel harvesting device	2,0	1,1	0,309	2,4	0,00252	1,2	0,00588	1,1
Central venous catheter insertion set	2,5	1,4	0,0663	0,5	0,00215	1,0	0,00528	1,0
Pleural drainage system	2,4	1,4	0,0486	0,4	0,00328	1,5	0,00684	1,3
Urine collection container	2,0	1,1	0,0852	0,7	0,00226	1,1	0,00496	1,0
Pefusionist disposables (others)	4,0	2,3	0,27	2,1	0,00416257	2,0	0,01001174	1,9
"CABG tray"	5,8	3,3	0,20	1,5	0,00762947	3,6	0,01735379	3,4
"Wound drain tray"	4,6	2,7	0,13	1,0	0,00744573	3,5	0,02223231	4,3
Anaesthesia disposables (others)	4,3	2,4	0,17	1,3	0,00766033	3,6	0,01827664	3,5
General surgical disposables	3,7	2,1	0,19	1,5	0,00396155	1,9	0,00939209	1,8

"Endoscopic vessel tray"	1,8	1,0	0,23	1,8	0,0002366<br>4	0,1	0,0023323<br>3	0,5
"Kidney tray"	1,3	0,7	0,11	0,9	0,0020372<br>8	1,0	0,0036401<br>5	0,7
ICU disposables (others)	5,9	3,3	0,3	2,1	0,00647	3,0	0,01488	2,9
Syringes	1,82	1,0	0,05	0,4	0,00227	1,1	0,00501	1,0
Nitrile gloves	1,66	0,9	0,11	0,9	0,00226	1,1	0,00514	1,0
Volumetric exerciser	1,5	0,9	0,03	0,2	0,00177	0,8	0,0039	0,8
Protective aprons	1,23	0,7	0,0312	0,2	0,00152	0,7	0,00351	0,7
Ventilator disposables	1,09	0,6	0,04	0,3	0,00101	0,5	0,00236	0,5

Table F6. Snapshot of OR HVAC contribution analysis

				% of total	% of OR energy
**Total impact of OR energy use**		47,603197	kg CO2 eq	11,5034224	100%
total impact of HVAC electricity		32,9381685	kg CO2 eq	7,95958445	69,1931857
total impact of HVAC natural gas		4,46748262	kg CO2 eq	1,07957749	9,38483737

Values for HVAC were calculated based on annual averages, excluding end-of-pipe heating of air for individual ORs..

Table F7. Snapshot of ICU HVAC contribution analysis

**ICU stay duration (median)**		22,1	hours		
				**% of total**	**% of ICU energy**
**Total impact of OR energy use**		31,260967	kg CO2eq	7,55428483	100
total impact of HVAC electricity		22,1598269	kg CO2eq	5,35497333	70,886569
total impact of HVAC natural gas		6,82831999	kg CO2eq	1,65007929	21,8429583

Values for HVAC were calculated based on annual averages, excluding end-of-pipe heating of air for individual ICU patient rooms.

Table F8. Contribution analysis for reusables in OR and ICU

Impact category	Unit	Total	Top	PP wrapper production	PP production %	PP wrapper incineration	PP incineration %	Paper wrapper	Paper wrapper %	Electricity for sterilisation	Electricity %	Natural gas production for sterilisation	Natural gas production %	Natural gas incineration for sterilisation	Natural gas incineration %	Detergent for sterilisation	Detergent %	Steel production + metal working	Steel %	Water for sterilisation	Water %
Global warming	kg CO_2 eq	21,345325	0,293494	1,1957532	5,6	0,97126511	4,6	0,28713452	1,3	5,015803	23,5	1,9958383	9,4	9,64	45,2	0,85498029	4,0	0,7519971	3,5	0,33905946	1,6
Stratospheric ozone depletion	kg CFC11 eq	5,91E-06	1,37E-07	2,15E-07	3,6	7,00E-08	1,2	2,62E-07	4,4	1,74E-06	29,4	8,45E-07	14,3	x	x	2,32E-06	39,3	1,95E-07	3,3	1,32E-07	2,2
Ionizing radiation	kBq Co-60 eq	0,33119889	0,01728759	0,04833092	14,6	9,20E-05	0,0	0,0552493	16,7	0,02174993	6,6	0,01236601	3,7	x	x	0,01431941	4,3	0,04516846	13,6	0,11663527	35,2
Ozone formation, Human health	kg NOx eq	0,02070964	0,00069856	0,00278322	13,4	0,0001528	0,7	0,00120327	5,8	0,00830667	40,1	0,00368953	17,8	x	x	0,00126892	6,1	0,00179798	8,7	0,0008087	3,9
Fine particulate matter formation	kg PM2.5 eq	0,00958568	0,00058652	0,00149645	15,6	2,25E-05	0,2	0,00040045	4,2	0,00242749	25,3	0,00080658	8,4	x	x	0,00094487	9,9	0,00234437	24,5	0,00055645	5,8
Ozone formation, Terrestrial ecosystems	kg NOx eq	0,02214134	0,00073335	0,0029354	13,3	0,0001538	0,7	0,00127903	5,8	0,00865356	39,1	0,00429771	19,4	x	x	0,00137034	6,2	0,00187153	8,5	0,00084663	3,8
Terrestrial acidification	kg SO2 eq	0,02087366	0,00145608	0,0034666	16,6	6,52E-05	0,3	0,00118669	5,7	0,00676796	32,4	0,00186879	9,0	x	x	0,00219745	10,5	0,00264023	12,6	0,00122465	5,9
Freshwater eutrophication	kg P eq	0,01113832	0,00010894	0,0002701	2,4	2,49E-05	0,2	0,00076603	6,9	0,0001293	1,2	0,00014033	1,3	x	x	0,00916038	82,2	0,00031264	2,8	0,00022568	2,0
Marine eutrophication	kg N eq	0,00124955	2,48E-05	2,34E-05	1,9	1,50E-06	0,1	0,00011448	9,2	1,68E-05	1,3	1,55E-05	1,2	x	x	0,00099808	79,9	3,30E-05	2,6	2,20E-05	1,8
Terrestrial ecotoxicity	kg 1,4-DCB	41,994459	3,7825008	3,0752597	7,3	2,8366482	6,8	1,2004118	2,9	6,151278	14,6	2,101051	5,0	x	x	2,1513348	5,1	19,514677	46,5	1,1812984	2,8
Freshwater ecotoxicity	kg 1,4-DCB	0,31013491	0,05404807	0,03667186	11,8	0,06897495	22,2	0,01147993	3,7	0,00126149	0,4	0,02738736	8,8	x	x	0,03105255	10,0	0,06171864	19,9	0,01754008	5,7

Marine ecotoxicity	kg 1,4-DCB	0,41988353	0,07018795	0,04815561	11,5	0,09932735	23,7	0,01546963	3,7	0,00572179	1,4	0,03870015	9,2	x	x	0,02945244	7,0	0,08878156	21,1	0,02408705	5,7
Human carcinogenic toxicity	kg 1,4-DCB	1,6125915	0,03460602	0,04680413	2,9	0,00487577	0,3	0,03012498	1,9	0,02607869	1,6	0,14357756	8,9	x	x	0,02779835	1,7	1,018487	63,2	0,28023907	17,4
Human non-carcinogenic toxicity	kg 1,4-DCB	5,5972288	0,83467945	0,72839381	13,0	0,55489368	9,9	0,34073048	6,1	0,73628582	13,2	0,43267115	7,7	x	x	0,54872846	9,8	1,0148082	18,1	0,40603775	7,3
Land use	m2a crop eq	1,3864163	0,00825788	0,01408966	1,0	0,00011515	0,0	0,6550948	47,3	0,07863593	5,7	0,03138439	2,3	x	x	0,565636	40,8	0,02400314	1,7	0,00919938	0,7
Mineral resource scarcity	kg Cu eq	0,08409286	0,00719813	0,00238946	2,8	7,13E-05	0,1	0,00073131	0,9	0,00592768	7,0	0,00619654	7,4	x	x	0,00157087	1,9	0,05598323	66,6	0,0040243	4,8
Fossil resource scarcity	kg oil eq	7,6010415	0,08858248	0,72457731	9,5	0,00214671	0,0	0,07290678	1,0	1,8149621	23,9	4,5682612	60,1	x	x	0,06605385	0,9	0,17485391	2,3	0,08869718	1,2
Water consumption	m3	1,1296722	0,00438735	0,0041735	0,4	5,57E-05	0,0	0,00541493	0,5	0,01747691	1,5	0,00559417	0,5	x	x	0,02744504	2,4	0,0048055	0,4	1,0603192	93,9

Table F9. Sensitivity analysis of data choices (based on identified hostpots or minor contributions)

	Category	*Explanation*	*Type*	*Source*	*Effect*	*Further information*
1	Overall	Analysis method ReCiPe 2016 (H) vs adjusted PEF (SimaPro/PRé Sustainability)	Scientific basis for characterisation (PEF based on more recent characterisation factors than ReCiPe 2016)	suggestion LCA-expert	Difference in total global warming -2% (406 kg CO2eq vs 414 kg). Only minor variations in identified hotspots and no alterations of conclusions. Similar most contributing processes (and life cycle stages): i.e. electricity use, car transportation, production of polycarbonate, polyvinylchloride, and polypropylene plastics.	
2	Overall	Methodological choice ReCiPe 2016 (H) vs perspectives (I) and (E) - analysing differences on endpoint level (damage to human health, ecosystems, and resource scarcity)	Methodological/perspective choice within ReCiPe 2016, differentiating in weight for short term effects (I) or focus on long term effects (E)	suggestion LCA-expert	Conclusion: the (I) perspective emphasizes fine particulate matter formation as hotspot for damage to human health on the shorter term, for ecosystem damage no new hotspots are identified; the (E) perspective emphasizes non-carcinogenic and carcinogenic toxicity as the main hotspots for damage to human health; for ecosystem damage these are marine ecotoxicity and global warming.	Individualist (I) = 0,000143 DALYs; 9,41E-7 species*year ecosystem damage; 52.6 USD2013 resource scarcity; fine particulate matter formation causes most damage to human health (60%), then global warming (29%); for ecosytem damage none of the midpoint contribute more than 7% individually (global warming > terrestrial acidification > land use)<br><br>Egalitarian (E) = 0,035 DALYs; 2,49E-5 species*year ecosystem damage; 53,3 USD2013 resource scarcity; human non-carcinogenic (66%) and carcinogenic toxicity (18%) cause most damage to human health, global warming (15%); for ecosystem damage marine ecotoxicity (56%) and global warming (41%) cause most damage
3	Transportation	Dutch average car transportation mix for employees and patients (Ecoinvent) rather than the Netherlands-specific STREAM transportation data	Alternative data choice	own hotspot identification	Difference of total global warming +9% (33,5 & 80,2 kgCO2eq vs 22,6 & 54,6 kg CO2eq). Hotspot identification of employee transportation 18% (formerly 13%) and patient transportation 7% (formerly 6%). **Most notably,** the environmental impact of land use is 12% lower in the Ecoinvent transportation mix scenario than in the STREAM scenario. Explained by the use of larger percentages of biofuel (E10 rather than E5 gasoline) for transportation.	
4	Energy	Dutch average electricity mix for OR and ICU (Ecoinvent) rather than the Netherlands-specific energy mix based on STREAM data CE Delft (2021): the NL mix contains more fossil fuel based electricity generation	Alternative data choice	own hotspot identification	Total GWP 433 kg CO2eq vs 414 kg (5% increase). Energy OR 60 (14%) vs 48 kg (12%). Energy ICU 31 (7%) vs 27 kg (6%) CO2 eq. Reusables 23 kg (5%) vs 21 kg (5%). **All impacts increase when selecting the Ecoinvent mix, no shift in hotspots.** No large differences in other hotspots, except for small increase in the impact of reusables due to the energy requirements for washing/desinfecting	

					and sterilisation. Difference for linen (due to energy required for washing process) is smaller than difference for sterilisation of reusables.	

Table F10. Sensitivity analysis of assumptions (based on identified hostpots or minor contributions)

	Category	*Explanation*	*Type*	*Source*	*Effect*	*Further information*
1	Medication	Medication spillage at OR and/or ICU not considered: 85% noradrenaline remaining in syringe, 80% (fenyl)efedrine, 60% propofol/rocuronium, 55% sufentanil, 45% remifentanil (*see "source" for origin of data, no hospital-specific data available)*. For other medication that is administered using syringes (e.g. midazolam), we assumed a similar spillage to propofol/rocuronium (60%) for the sensitivity analysis.	Underestimation of attributed impact to care pathway	Medical Delta, 4-day medication audit at LUMC (Leiden) OR	Difference of total global warming <1%. Contribution of medication 4% vs 4% (19.2 kg CO2 eq vs 16.6kg CO2 eq). Other environmental impacts not included in the analysis and can therefore not be compared. Conclusion: minor impact on total carbon footprint of care pathway.	Spillage only applies to part of medication. E.g. albumin (largest uknown impact) comes in preset size (and therefore spillage does not occur). Only API production is considered. Possibility to assume additional impact for 'carrier substances' --> pharmaceutical company document suggests that majority of impact (for small molecule drugs) is caused by API synthesis. Human albumin is a by-product of production of packed cells. No information available regarding the environmental impact of this process.<br><br>On the contrary, e.g. custodiol is currently entered as 'fluid therapy' and a regular crystalloid (NaCl) is used as proxy. However, custodiol mainly consists of 'histidine', which is biosynthesized e.g. by bacteria, making it possible that the impact would be higher.
2	Reusables	Approximately 70% slot occupancy for washer/desinfector and steam steriliser. Reference calculation assumed 100% usage of slots, wherein most CABG trays are a 'big set', meaning that they will be divided over two separate nets prior to washing and sterilisation. Assuming 18 slots (steriliser 12 slots occupied) and assuming 12 slots (washer/desinfector 8 slots are occupied)	Underestimation of attributed impact to care pathway	Occupancy data in Rizan study; consultation of sterilisation expert at university hospital.	Difference of total GWP 1%, same for land use (+1%), **mainly difference for environmental impact water use (+8%)**. Reusables contribute 18% of water use vs 11% in reference scenario. Contribution of reusables for GWP 3.5% vs 2.4%.	
3	Reusables	Different sterilisation process available, specifically measured in other Dutch university medical centre (UMCU), which has a relatively 'new' sterilisation department. Impact of alternative process likely to be an underestimation based on report of original authors, therefore multiplied by factor 1,5 to get closer to Rizan process. Comparison for other environmental impacts than global warming potential.	Overestimation of attributed impact to care pathway	Most recent, also in the Netherlands (Schmidt, 2023); others, more extensively modelled (Rizan 2022 and McGain 2016)	Difference of total GWP <1% smaller; other environmental impacts: land use increased +3% and **water use decreased by 7% compared to the reference scenario**. Reusables contribute 8.3% of land use and 3.7% of water use compared to 5.5% and 11% in the reference scenario.	
4	Transportation	Survey of OR staff travel and general ICU travel survey may overrepresent local	Underestimation due to differences in preferred	own hotspot identification, staff survey at LUMC	Difference of total GWP <1%; other environmental impacts	

		hospital. Based on data of other academic hospital, assumed 35.6km per employee (17.8km one way) and that 50% comes by car (fewer during day shifts, more during night shifts).	means of travel or differences in the actual number of healhcare staff responsible for one patient	(Leiden) regarding travel info 2021	have similar (minor) differences to the reference situation. Employee commute 59kg CO2 eq vs 55 in reference situation. Conclusion: minor impact on total environmental impacts of care pathway.	
5	Disposables	Waste processing --> 'incineration of medical waste' process which contains energy recovery (cogeneration recovery of 36%) and average caloric content of 19.1 MJ/kg of medical waste. However, process does not include plastic-specific inputs, which makes it susceptible to underestimation of waste incineration impacts	Overestimation of the environmental impact of the end of life phase	RIVM incineration process [van Bodegraven & Pieters, RIVM, 2023; het effect van persoonlijke beschermingsmiddelen op het milieu]	Difference of total GWP 3% reduction; other environmental impacts: e.g. 1% reduction for land use. Mainly for the disposables the environmental impact contributions for GWP reduce: OR disposables XX% vs 40% and ICU disposables X% vs 3%.	

Note to reader: variance of energy use in ICU and OR were already included in the uncertainty analysis (Monte Carlo) by predefining possible ranges for electricity using the Pedigree Matrix. Therefore, no separate sensitivity analysis has been performed to test alternative assumptions for the **amount** of energy used.

Table F11. Detailed scenarios of impact mitigation possibilities

Category	Scenario	Changes to LCA-model	Global warming change
Energy use OR + ICU	Hospital runs entirely on renewable energy, rather than (partially) fossil fuel powered [a]	Replaced all energy use of the (tailored) 'Dutch electricity mix' (2021) with the (tailored) Dutch green energy mix (2021).[14] Effectively, this means that instead of 66% non-renewable energy (0.374 kg CO_2 eq/kWh - mainly natural gas, coal, and biomass), 100% renewable energy is used.	- 16% (66 kg CO_2eq)
Employee commute	Public transportation is used to commute to the hospital instead of commute by car [a]	Replaced all car transportation in the model with public transportation using train (50%) or bus (50%) from the (tailored) Dutch transportation mix (2021).[15] Increased travel distances by 20% to account for an increase of commute distance due to more indirect travel.	- 8% (35 kg CO_2eq)
Employee commute	All-electric car transportation instead of fossil fuel powered cars [a]	Replaced all car transportation in the model with electric car transportation from the (tailored) Dutch transportation mix (2021).[15] Distances were left unaltered. Electric cars were powered by the regular (tailored) 'Dutch electricity mix' (2021).[14]	- 6% (23 kg CO_2eq)
Disposables OR	Reusable surgical drapes instead of disposable surgical drapes *[b]*	Calculated a 50% environmental impact reduction for the total global warming of surgical drapes, based on a previous	- 4% (18 kg CO_2eq)

		review and comparative LCA (2012, 2022).[25,26]	
Disposables OR	Reusable surgical gowns instead of disposable surgical gowns [b]	Calculated a 66% environmental impact reduction for the total environmental impact of surgical gowns, based on a previous comparative LCA (2018).[26]	- 2% (7 kg CO_2eq)
Energy use OR	Reduced HVAC air refreshment rate in OR (-16% energy use) [c]	Lowered the parameters for the amount of fresh outside air (1,700 m^3/h) and recirculated air (6,800 m^3/h) in close consultation with a hospital HVAC specialist and current Dutch infection prevention standards. Results in a lowered electricity and natural gas demand, compared to Table E6.	- 1 % (5 kg CO_2eq)
Energy use OR + ICU	Loosen HVAC setpoints for relative air humidity (30% lower boundary vs 40%) [c]	Reduced amount of natural gas required and incinerated, based on a calculated 75% reduction of natural gas demand using annual hospital HVAC data. The 30% relative humidity setpoint results in a lower threshold of 5g/kg instead of 7g/kg.	- 1 % (4 kg CO_2eq)
Disposables ICU	Reduction of ICU disposables (others), glove usage, and protective aprons (-30%) [d]	Calculated a 30% impact reduction based on the total environmental impact of nitrile gloves, protective aprons, and “ICU disposables (others)” (Table F5).	- 1% (3 kg CO_2eq)

[a] Hypothetical scenarios based on the environmental hotspots identified in this study. Scenarios for energy use and employee commute are in line with the studied hospital’s environmental sustainability policies.
[b] Scenarios based on previous studies comparing disposable and reusable surgical drapes and gowns.
[c] Scenarios based on calculations regarding HVAC energy reduction in close consultation

with in-hospital engineers.
[d] *Scenario based on an assumed 30% reduction in weight (or quantity) of disposables.*
Numerical references correspond to order in the manuscript. Legend: OR = operating room; ICU = intensive care unit; LCA = life cycle assessment; HVAC = heating, ventilation, and air conditioning system.

Note to reader: while the table above only includes the hypothetical reduction of global warming incurred by a CABG trajectory (considering that this was the environmental impact causing most harm to human health and ecosystems), the scenarios that reduce non-renewable energy use, individual car commute, and disposable product use also benefit other environmental impacts of fossil-based resources (e.g. fine particulate matter formation). A detailed comparison of reusable surgical drapes and gowns (based on multiple environmental impact categories) can be found in the referenced LCA-studies: Bijleveld M, Uijttewaal M. LCA herbruikbare en eenmalige ok-jassen en afdekmateriaal. Delft: CE Delft;2022 and Overcash M. A comparison of reusable and disposable perioperative textiles: sustainability state-of-the-art 2012. Anesth Analg. 2012;114(5):1055-1066.

Supplement S7 – Acknowledgements

We thank Radboudumc staff for their participation in the data collection and interest in the study. Specifically, we thank Michiel Brands, Aat Builtjes, and Martin Janssen for their help in quantifying energy use of the OR and ICU, and Philips for their cooperation to include the impact of long-standing medical devices in this analysis. To all 'green' professionals involved along the way: next to your ongoing sustainability initiatives, we hope the final product will have been worth your efforts.
