# Supplementary figures and images for: Assessing the environmental impact of coronary artery bypass grafting to decrease its footprint
Source: Eur J Cardiothorac Surg. 2025 Feb 17;67(2):ezaf054. doi: 10.1093/ejcts/ezaf054 (PMC11879344; doi:10.1093/ejcts/ezaf054)

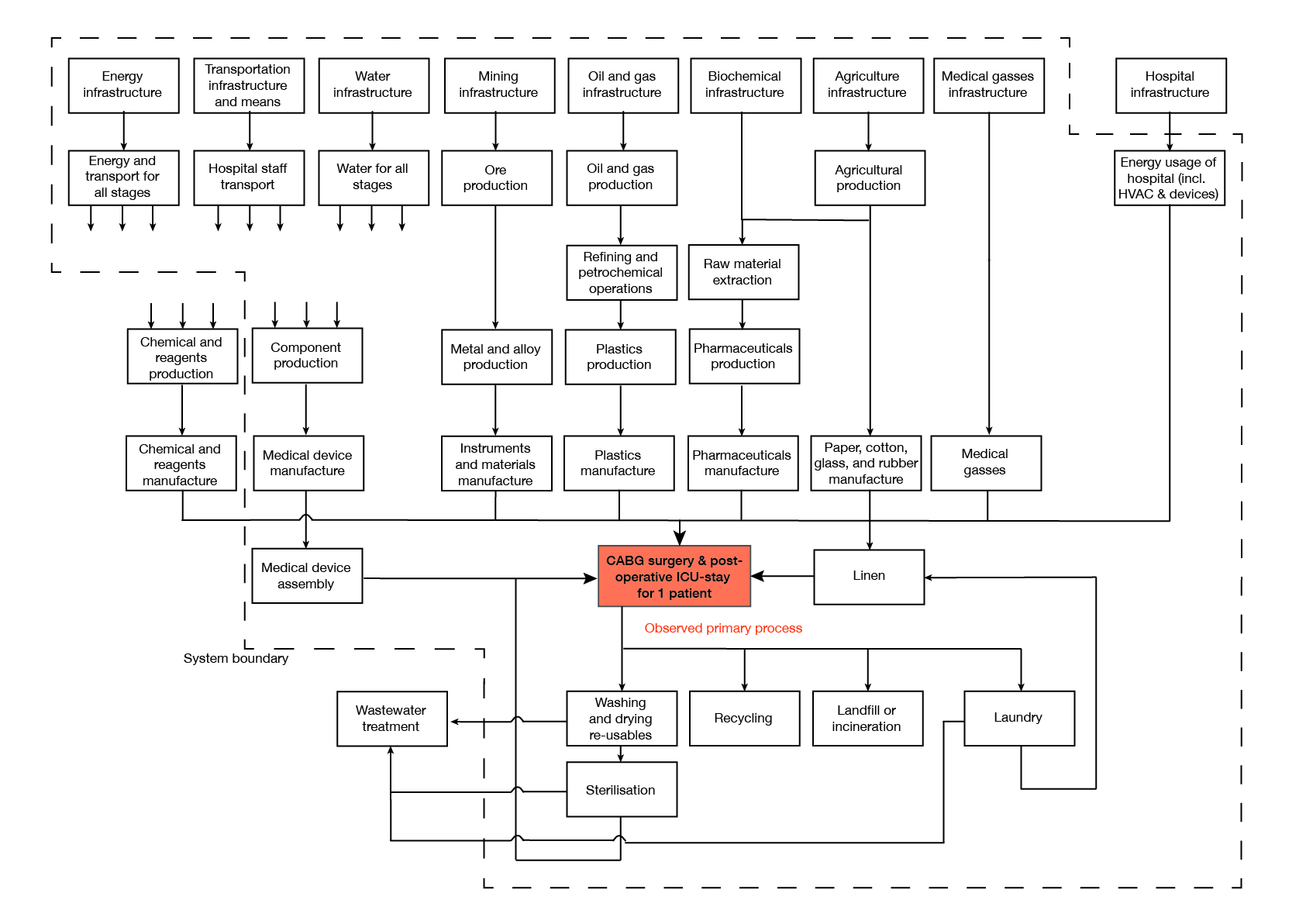

Supplement: ezaf054_Supplementary_Data [file ezaf054_supplementary_data.zip › Supplement_S1_-_CABG_OR_ICU_system_boundaries_LCA_v240124.jpg]
